# Mechanically-adaptive, resveratrol-eluting neural probes for improved intracortical recording performance and stability

**DOI:** 10.1038/s41528-025-00440-5

**Published:** 2025-07-09

**Authors:** Natalie N. Mueller, Mali Ya Mungu Ocoko, Youjoung Kim, Kate Li, Kaela Gisser, Gabriele Glusauskas, Isabella Lugo, Peter Dernelle, Anna Clarissa Hermoso, Jaime Wang, Jonathan Duncan, Lindsey N. Druschel, Francine Graham, Jeffrey R. Capadona, Allison Hess-Dunning

**Affiliations:** 1 Advanced Platform Technology Center, VA Northeast Ohio Healthcare System, Cleveland, OH USA; 2 Department of Biomedical Engineering, Case Western Reserve University, Cleveland, OH USA

**Keywords:** Electrophysiology, Bioinspired materials, Biomaterials, Implants

## Abstract

Intracortical microelectrodes are used for recording activity from individual neurons, providing both a valuable neuroscience tool and an enabling medical technology for individuals with motor disabilities. Standard neural probes carrying the microelectrodes are rigid silicon-based structures that can penetrate the brain parenchyma to interface with the targeted neurons. Unfortunately, within weeks after implantation, neural recording quality from microelectrodes degrades, owing largely to a neuroinflammatory response. Key contributors to the neuroinflammatory response include mechanical mismatch at the device-tissue interface and oxidative stress. We developed a mechanically-adaptive, resveratrol-eluting (MARE) neural probe to mitigate both mechanical mismatch and oxidative stress and thereby promote improved neural recording quality and longevity. In this work, we demonstrate that compared to rigid silicon controls, highly-flexible MARE probes exhibit improved recording performance, more stable impedance, and a healing tissue response. With further optimization, MARE probes can serve as long-term, robust neural probes for brain-machine interface applications.

## Introduction

Neural probes with recording microelectrodes are powerful tools for advancing our understanding of neural connectivity and restoring motor and communication function in individuals with neuromusculoskeletal conditions, such as spinal cord injury, limb loss, or neurodegenerative disorders^1–8^. Placing the recording microelectrodes into the cortex facilitates a close interface with neurons for extracellular recording of single-unit action potentials produced by individual neurons. Arrays of microelectrodes can record spatially resolved information-dense signals that encode the activity of a population of neurons. As part of a brain-machine interface, recorded signals can be decoded and translated into control signals for assistive technologies or prosthetic limbs^2^. Signals recorded from intracortical microelectrodes can also be used to control functional electrical stimulation systems for restoring native arm and hand control^4^. Additionally, individuals with a neurodegenerative disorder such as Parkinson’s disease or essential tremor can utilize deep brain stimulation with intracortical microelectrodes to restore motor control^8,9^.

Successful implementation of neural probes for intracortical recording requires reliable chronic use—potentially for decades. Unfortunately, recording performance begins to degrade within only weeks after implantation, characterized by reduced recording performance in the form of decreased active electrode yield, signal-to-noise ratio, and signal amplitude^10–19^. Performance degradation, and ultimately failure, is largely attributed to a neuroinflammatory response to the probe that is initiated with insertion-related cellular and vascular damage^1,12^, followed by a foreign body response cascade that contributes to chronic inflammation^20–23^. The neuroinflammatory response causes neural probe encapsulation by glial scar tissue, as well as neuronal degradation near the probe^24–30^. Additionally, activated microglia and macrophages recruited to the implant site release pro-inflammatory and cytotoxic soluble factors, such as reactive oxygen species^31–36^. The upregulation of reactive oxygen species in the tissue can lead to a state of oxidative stress, culminating in neuronal death as well as oxidative damage to the implanted probe microelectrodes and insulation layers^37–43^. In turn, high-quality signals with distinct single units cannot be recorded as healthy neurons become too distant from the recording microelectrode^44–47^.

Standard neural implants for intracortical recording are made from rigid materials, such as silicon^48,49^. The rigid materials aid in placing the microelectrodes into the cortex without buckling, but then have a 10^7^-fold modulus mismatch with the surrounding tissue that can further exacerbate the neuroinflammatory processes and prevent a healing response. In response, mechanically-flexible neural probes from materials with lower elastic moduli and flexural stiffness have been developed to attenuate mechanical mismatch in intracortical microelectrode failure^50–52^. Flexible, compliant polymers and in situ softening substrates have been shown to reduce astrocytic glial scarring and blood-brain barrier permeability while improving neuronal density^50,52–62^. However, implanting highly flexible probes can be challenging, often requiring additional reinforcement structures or strategies that complicate the surgical insertion process.

In this work, we evaluated the performance of a mechanically-adaptive, resveratrol eluting (MARE) neural probe architecture that addresses both mechanical mismatch and oxidative stress as neural probe failure mechanisms. The probes used a mechanically-adaptive polymer nanocomposite (NC) structure^63,64^. The stiffness of the material is governed both by temperature and by hydrogen bonding interactions between the cellulose nanocrystals, which are disrupted by water. The cellulose nanocrystals also promote water absorption, plasticizing the polymer and lowering its glass transition temperature to below body temperature. The NC is rigid (elastic modulus *E*_dry_ = 5 GPa) when dry to facilitate probe insertion^63^. After deployment, the NC dramatically softens within minutes (*E*_implanted_ = 10 MPa) as it absorbs water under physiological temperatures^63,64^. After softening, the NC much more closely matches the brain’s elastic modulus (*E*_brain_ ~ 10 kPa), reducing modulus mismatch with the brain by a factor of 10^5^ compared to silicon. As a result, stress and strain on the tissue surrounding the NC probes is significantly reduced compared to the rigid silicon control^65^. Previous immunohistochemistry results suggest that NC, along with other flexible, mechanically-compliant materials, have distinct advantages in the neuroinflammatory response compared to rigid controls^66,67^. NC probes showed higher neuronal density near the implant by 2 weeks with no neuronal loss around the implant by 16 weeks, less glial scarring than the rigid controls, and essentially complete blood-brain barrier healing by 16 weeks^66^.

Oxidative stress can be mitigated through systemic or local delivery of antioxidants such as resveratrol and curcumin, which improve the neuroinflammatory response by reducing oxidized proteins, activated microglia, and macrophages, as well as increasing neuronal density around the implant site^40,41,68–71^. Resveratrol, a naturally occurring polyphenol, mitigates oxidative stress through direct neutralization of reactive oxygen species (ROS) and reactive nitrogen species (RNS) and through the activation of endogenous pathways that promote the production of antioxidant proteins^72–75^. As the mechanically-adaptive properties of NC are reliant upon its small molecule permeability, therapeutic agents can be incorporated into the NC for elution into tissue after implantation. Nguyen et al. showed that incorporating resveratrol into the NC improved immunohistological markers of neuroinflammation^68^. At the 2-week time point, resveratrol-eluting NC probes showed a higher neuronal density within the critical 50 µm of the implanted probe compared to non-eluting NC probes^68^. Therefore, we have fabricated MARE probes from resveratrol-loaded NC, yielding microfabricated microelectrode arrays for single-unit neural recordings on an antioxidant-eluting substrate that softens after implant.

We hypothesized that the combination of mechanical flexibility from the mechanically-adaptive NC and oxidative stress reduction via local resveratrol delivery would enhance single-unit neural recording performance by improving integration with cortical tissue. Here, we evaluated the hypothesis by comparing the chronic neural recording performance of MARE probes and commercially-available, industry-standard, silicon-based NeuroNexus probes in rats. Recorded units were classified in terms of repolarization duration to determine the relative proportion of units recorded from putative inhibitory or excitatory neurons for each probe type. At either an acute (4-week) or chronic (12-week) time point, the tissue surrounding the implanted probes was analyzed to quantify gene expression related to oxidative stress and neuroinflammation. While immunohistochemistry (IHC) is a well-established method for evaluating the cellular responses to implanted neural probes^66,68,76,77^, it is limited by the number of markers that can be assessed simultaneously. In contrast, gene expression profiling enables a broader and more mechanistic view of the tissue environment. Here, we used a custom panel of 146 genes related to neuroinflammation, oxidative stress, and neuronal health, as well as 6 additional housekeeping genes^78^. This marks the first study evaluating the chronic recording profile of MARE neural probes, as well as the first comparison of MARE probes and NeuroNexus probes in terms of recording performance and neuroinflammation and oxidative stress responses. Our results suggest that MARE neural probes are a promising platform for promoting implant-tissue integration for improved chronic neural recording.

## Results

### MARE neural probe design

**Fig. 1:**
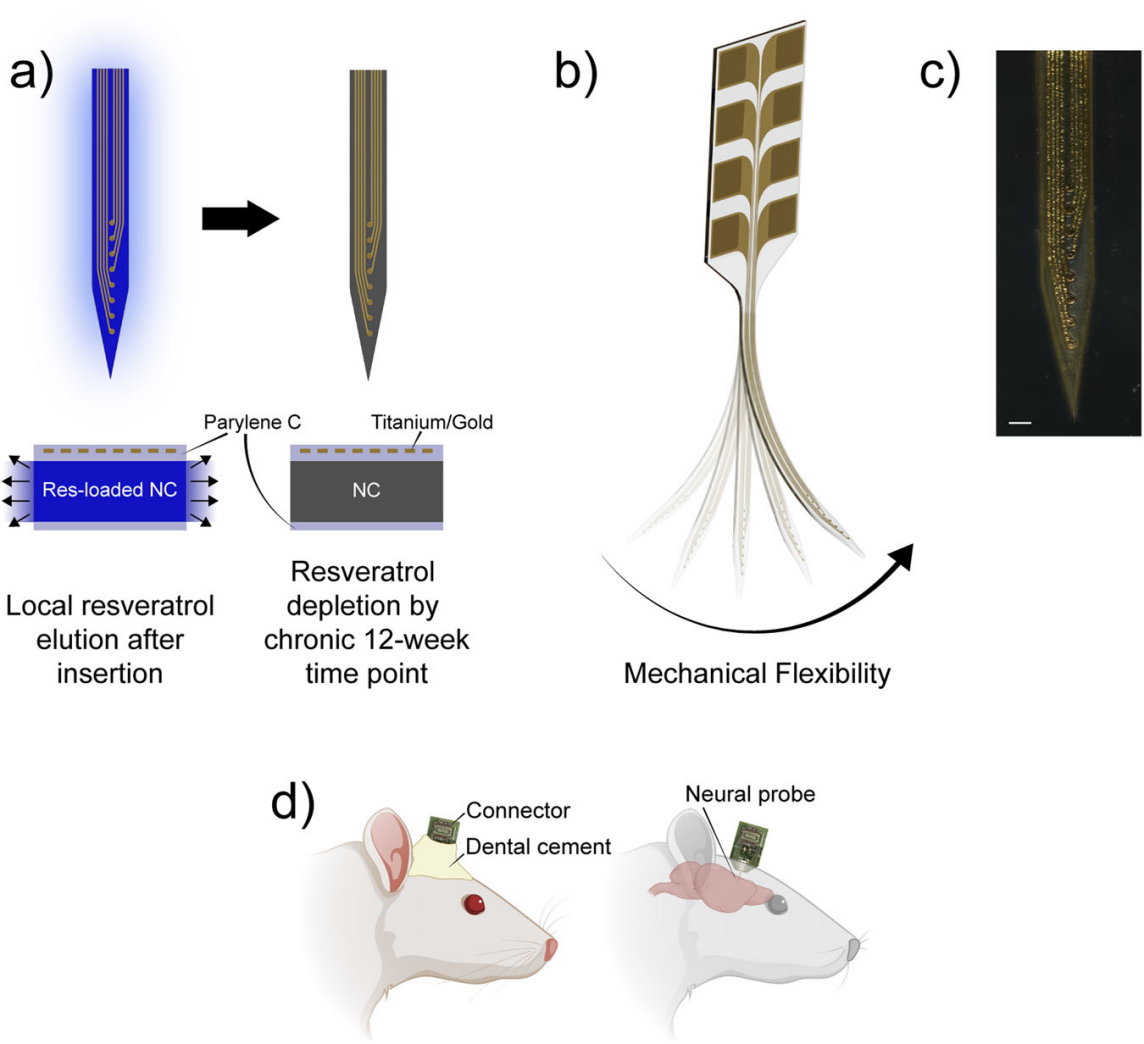
Overview of MARE probe design, structure, and function. **a** A plan-view (top) and cross-sectional view (bottom) of the MARE probes, showing that the NC is initially loaded with resveratrol. By the chronic end point, the resveratrol has been depleted from the shank by eluting through the probe sidewalls; **b** After insertion while rigid, the nanocomposite (NC) shank absorbs fluid and becomes mechanically-flexible; **c** Microscope image of the recording microelectrode sites on a MARE probe. Scale bar represents 100 µm. **d** Schematic of implanted neural probes showing dental cement securing the probe and attached printed circuit board in place (left) and how the probe is inserted into the rat’s brain (right) (Created in BioRender).

An overview of the mechanically-adaptive, resveratrol-eluting (MARE) probes used in this study is shown in Fig. 1. MARE neural probes in this study were 50 µm-thick single-shanks with a linear array of eight recording microelectrode sites^79^. The recording microelectrodes were each 30 µm in diameter and spaced 100 µm apart center-to-center. Each shank is made of layers of a resveratrol-doped mechanically-adaptive polymer nanocomposite (NC) based on polyvinyl acetate and tunicate-derived cellulose nanocrystals, Parylene C, and photolithographically-patterned thin-film gold traces with a titanium adhesion layer (Fig. 1a)^79,80^. Probes are packaged onto a custom printed circuit board that facilitates connection with external electronics. The use of Parylene C layers on the top and bottom surfaces serves to slow the resveratrol diffusion rate from the NC. In a previous study, we used NanoDrop spectrophotometry to determine that structures replicating the architecture and critical dimensions of MARE probes release resveratrol for up to 60 days with a Fickian diffusion profile^79^. After implantation, aqueous fluids can diffuse inward as resveratrol diffuses from the device to the local tissue, where it can scavenge reactive oxygen species. Additionally, as aqueous fluids are absorbed by the probe, the NC becomes 3 orders of magnitude more flexible (Fig. 1b).

A microscope image of the probe recording sites is shown in Fig. 1c. When the NC swelled, the MARE layers remained intact (Fig. S1) and no gross shank curling was observed, which can likely be attributed to dimensional changes primarily affecting the thickness of the NC film^81^. Mechanically-adaptive (MA) probes without resveratrol doping were also fabricated with an otherwise identical construction. The silicon-based control NeuroNexus probes (A1x16-3mm-100-177-Z16, NeuroNexus, Ann Arbor, MI, USA) are 15 µm-thick and have a similar linear array of 16 recording microelectrodes, each 15 µm in diameter and spaced 100 µm center-to-center. The shank is made from silicon and silicon-based insulators that cannot be loaded with resveratrol for local elution. The MARE and MA probes were packaged using a printed circuit board and connector configuration similar to that of the NeuroNexus probes, allowing for a consistent surgical procedure across groups. The printed circuit board extended above the closed skin and was secured with dental cement to provide electrical access to the probe via a connector, as shown schematically in Fig. 1d.

### In vivo neural recording performance

**Fig. 2:**
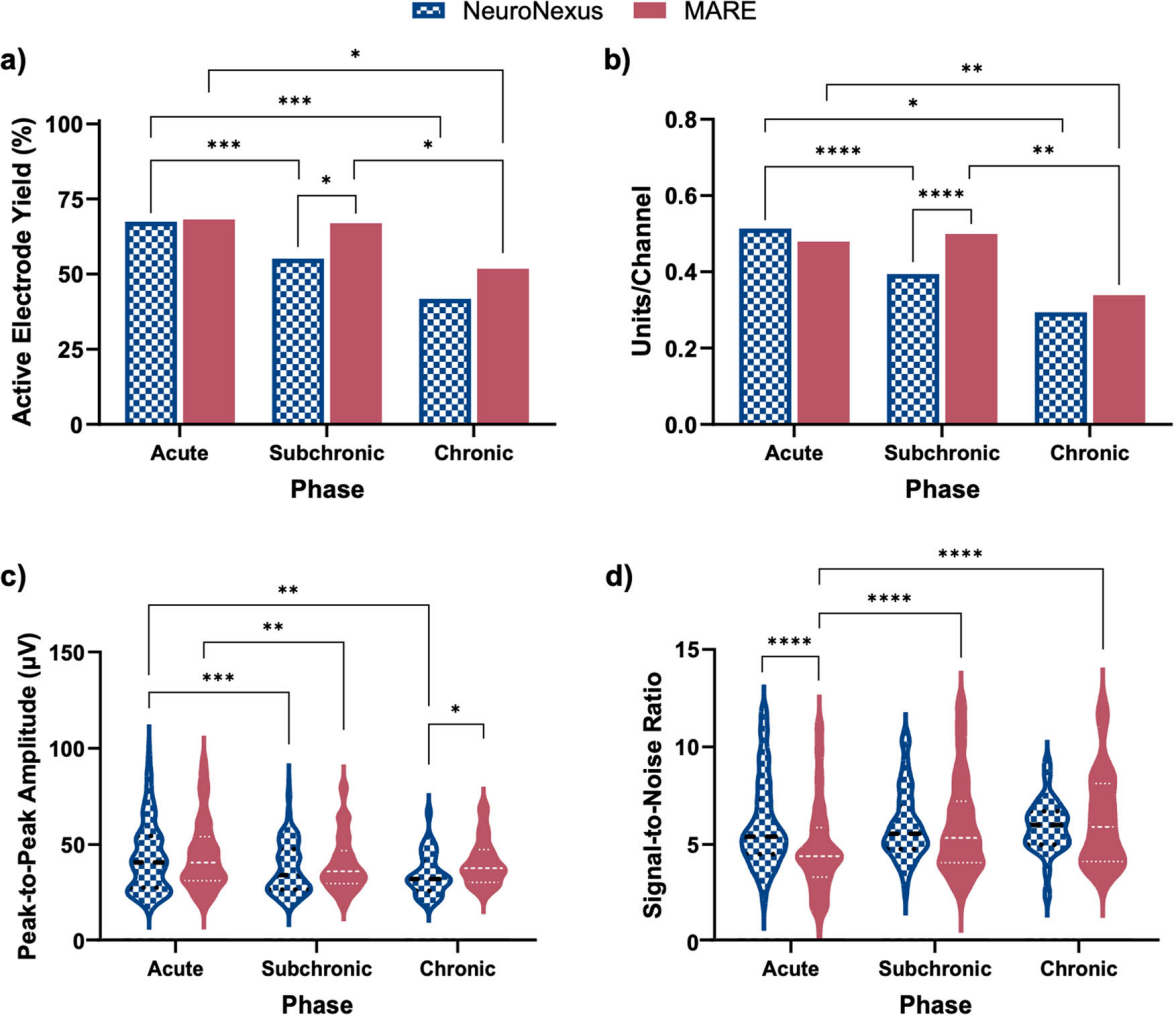
Recording metrics for NeuroNexus silicon control probes and MARE probes at the acute, subchronic, and chronic time points. **a** Active electrode yield, **b** number of distinct units per channel per group, **c** recorded single unit peak-to-peak amplitude, and **d** the signal-to-noise ratio of the recorded and isolated single units. Significance is denoted by * for *p* < 0.05, ** for *p* < 0.01, *** for *p* < 0.001, and **** for *p* < 0.0001.

The single-unit recording performance of the flexible MARE probes was compared to rigid silicon-based NeuroNexus probes across acute (weeks 0–5), subchronic (weeks 6–11), and chronic (week 12) phases using a set of single-unit recording summary metrics (Fig. 2). The phases are indicative of tissue response characteristics, with the acute phase marked by the dynamic environment caused by insertion-induced tissue damage and the activation of innate immune pathways^10,11,82,83^. The subchronic phase is characterized by a transition from acute to chronic neuroinflammation, as well as the development and maturation of the glial scar. The chronic phase is associated with a stabilization of chronic neuroinflammation and a mature glial scar. A total of twelve probes of each type were used in the study, with one single-shank probe implanted into the primary motor cortex of each Sprague-Dawley rat. Probes remained in place for either 4 (acute time point, five animals per probe type) or 12 (chronic time point, seven animals per probe type) weeks. One animal with a NeuroNexus probe and one animal with a MA probe implanted died before the 4-week time point and were therefore excluded from the results of the study, leaving four animals in the acute NeuroNexus and MA groups.

An active electrode is defined as a microelectrode channel that recorded a single unit action potential during the recording session. The active electrode yield (AEY), or the proportion of the electrodes recording single unit action potentials for each probe type and phase, indicated that the MARE probe experimental group performed similarly or better across all phases compared to the control NeuroNexus (Fig. 2a) and MA probes (Supplementary Fig. S2 and Table S1). To control for differences in recording depth, only the bottom 8 (of 16) recording channels on the NeuroNexus probes were included in all recording analyses. Channels that did not record a single unit during any recording session were omitted from the analysis. An electrode was considered to be active if units were recorded during either of two recording sessions each week. The total possible electrode count for each phase is the sum of the total electrode count across the number of phase weeks. NeuroNexus probes had a total of 387, 330, and 55 possible electrodes for the acute, subchronic, and chronic time points, respectively. MARE probes had a total of 428, 336, and 56 possible electrodes for the acute, subchronic, and chronic time points. AEY for NeuroNexus and MARE probes across time points is shown in Fig. 2a. During the acute phase, MARE and NeuroNexus probes had an AEY of 70%, similar to other studies characterizing AEY in intracortical microelectrodes^10^. During the subchronic phase, MARE probes maintained the acute phase AEY, while NeuroNexus probes had a 12% decrease (~1 channel per probe) in AEY. In the subchronic phase, MARE probes had a statistically significant higher AEY than the NeuroNexus probes. In the chronic phase, both groups exhibited reduced AEY; however, MARE probes had a 10% higher AEY with 5 additional channels with detectable units across all probes. Figure S2a shows the AEY of the MA probes. MA probes had a total of 384, 336, and 56 possible electrodes for acute, subchronic, and chronic phases, respectively. MA probes exhibited a statistically significant lower AEY (Fig. S2) compared to the MARE group in the acute time point. Table S1 shows the *p* value of all comparisons across groups and phases.

The number of individual single units recorded per the number of total channels is shown in Fig. 2b, with trends that are similar to the AEY. NeuroNexus probes exhibited a drop in units per channel across each phase, while MARE probes maintained the number of units per channel through the subchronic phase before showing a drop at the chronic time point. Figure S2b shows the units per channel of the MA probes, which experienced statistically significant declines through the chronic phase. Table S2 displays the *p* value of all comparisons across groups and phases for the number of units per channel.

Additional recording metrics of the average peak-to-peak amplitude and signal-to-noise ratio are shown in Fig. 2c, d, respectively. Signals detected from recording sites on NeuroNexus probes had similar peak-to-peak amplitudes compared to MARE probes, with an average of 44 ± 18 μV and 45 ± 18 μV, respectively, during the acute phase (Fig. 2c). From the acute phase to the subchronic phase, the average peak-to-peak amplitude for both probe types slightly decreased by ~ 4–6 µV. Overall, the MARE probes demonstrated improved stability in peak-to-peak amplitude compared to the NeuroNexus probes, which continued to decrease at the chronic phase. At the chronic time point, the MARE probes recorded statistically significantly higher peak-to-peak amplitudes than the NeuroNexus probes. Figure S2c shows the peak-to-peak amplitude from MA probes, which were similar to MARE probes at all phases. Table S3 shows the *p* value of all comparisons across groups and phases for peak-to-peak amplitude.

Figure 2d shows the signal-to-noise ratio (SNR) from NeuroNexus and MARE probes across the three phases. During the acute phase, signals from NeuroNexus probes initially had a higher average SNR of 6.1 ± 2.3. MARE probe SNR improved at the subchronic (6.0 ± 2.5) and chronic (6.3 ± 2.4) phases while the NeuroNexus SNR was stable with an SNR of 6.0 ± 1.8 in the subchronic phase and 5.9 ± 1.3 in the chronic phase. MA probes demonstrated similar trends to the MARE probes, shown in Fig. S2d. All signal-to-noise ratio averages are considered high for extracellular neural recording^17,58,84^, indicating that the NeuroNexus, MARE, and MA probes demonstrate good recording quality. Additionally, all probes have sufficiently close neurons to record from with high signal-to-noise ratios, indicating that neuronal populations are present around the devices. Table S4 displays the *p* value of all comparisons across groups and phases for signal-to-noise ratio.

### Repolarization rate analysis

**Fig. 3:**
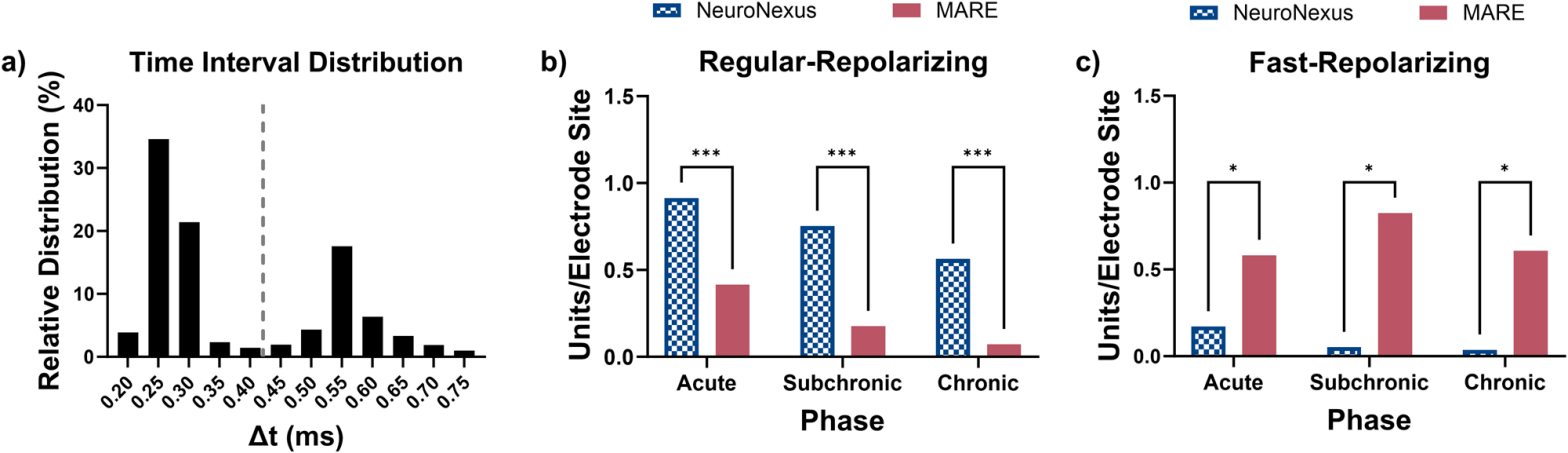
Repolarization rate analysis. **a** Histogram showing the relative distribution of the time interval between depolarization and repolarization for all units detected across NeuroNexus and MARE probes for the 12-week study. The dashed line indicates the threshold between fast-repolarizing and regular-repolarizing. **b** The number of regular-repolarizing units/electrode site was significantly higher for NeuroNexus probes compared to MARE probes for the acute, subchronic, and chronic phases. **c** The number of fast-repolarizing units/electrode site was significantly higher for MARE compared to NeuroNexus probes across all phases. Bars represent a ratio of units recorded divided by the total number of electrode sites, and therefore do not include error bars. Significance is denoted by * for *p* < 0.05 and *** for *p* < 0.001.

Repolarization rate analysis of the single unit action potential waveforms was used to classify recordings from putative inhibitory and excitatory neurons. The trough-to-peak time interval distribution for all units detected with MARE and NeuroNexus probes across all recording sessions is shown in Fig. 3a. The bimodal distribution supports classifying units with a time interval <0.40 ms as fast-repolarizing and units with a time interval >0.40 ms as regular-repolarizing, consistent with analyses from other groups^85–87^. The number of regular-repolarizing units/electrode recorded in NeuroNexus probes was significantly higher compared to MARE probes across all time points (Fig. 3b). Both groups experienced a decline in the number of regular-repolarizing units/electrode across the phases. However, MARE probes recorded significantly more fast-repolarizing units during all time phases compared to NeuroNexus probes (Fig. 3c). MARE probes demonstrated stability in the number of fast-repolarizing units/electrode across phases, while the NeuroNexus showed a decline. Supplementary Fig. S3 shows the time interval distribution and the number of units/electrode of fast-repolarizing and regular-repolarizing for all probe types, including MA probes. MA probes demonstrated similar trends to MARE probes, showing the preservation of fast-repolarizing neurons.

### In vivo impedance analysis

**Fig. 4:**
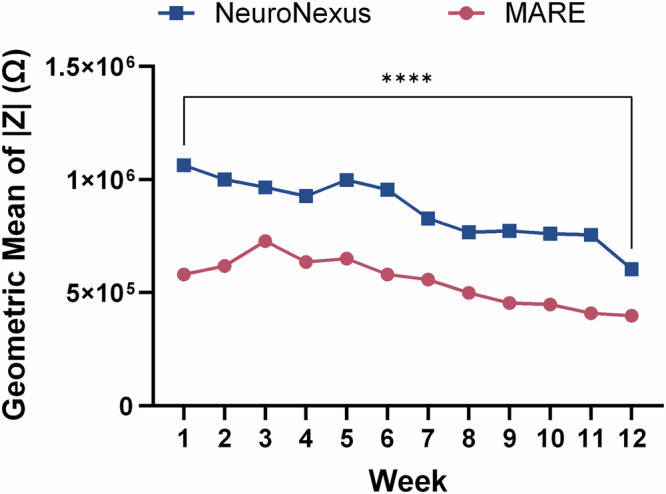
Geometric means of the impedance magnitude (|Z|) at 1 kHz by week for each probe type. NeuroNexus probes showed higher impedances and instability for the duration of study compared to MARE probes. See Table S5 for geometric standard deviation factors. Statistical significance is denoted by **** for *p* < 0.0001.

The in vivo impedance at 1 kHz was measured weekly to identify composition changes in the tissue environment and recording sites. Figure 4 shows the geometric mean of the impedance magnitude (|*Z*|) at the 1 kHz frequency across all channels for each probe type by week during the in vivo study. Additionally, Table S5 displays the geometric mean and geometric standard deviation (SD) factors for each probe type by week. The geometric SD factor describes the range of values about a geometric mean using a multiplicative factor. The data indicate that not only do NeuroNexus probes have a higher geometric mean impedance at all time points, which is at least partially attributed to the smaller site size, but they also experienced a steady decline in impedance throughout the study. MARE probes have a recording site surface area approximately 4x greater than NeuroNexus probes, contributing to the lower impedance. NeuroNexus probes have a geometric mean impedance magnitude at 1 kHz of 1064 kΩ during week 1, which decreases by 43% to 604 kΩ at week 12, a statistically significant decline with *p* < 0.0001. Meanwhile, MARE experienced a non-statistically significant percent decline of 32% (*p* = 0.15) between weeks 1 and 12. The stability of the MARE probe electrodes indicates durable, robust fabrication of the recording electrodes to maintain functionality in the harsh environment of the brain over time.

### Differential gene expression analysis

**Fig. 5:**
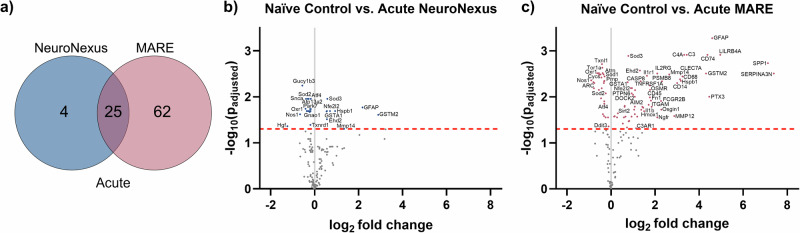
Differential expression for the NeuroNexus and MARE probes vs. naïve control at an acute time point of 4 weeks. **a** Venn diagram showing the number of significant differentially expressed genes between the groups, not accounting for upregulation vs. downregulation. **b**, **c** Volcano plots showing each probe type, where each point is a gene in the panel. The dashed red line shows the significance threshold of *p*_adjusted_ < 0.05. Significant differentially expressed genes are labeled and shown in color. Some significant genes were not labeled due to space limitations. Genes with a log_2_(fold change) >0 are upregulated from naïve control, and genes with a log_2_(fold change) <0 are downregulated from naïve control.

Differential gene expression analysis was conducted to determine how probe type affects oxidative stress and neuroinflammation around the implant site. All log_2_(fold change) values and significance estimates reflect group-level model outputs from the nSolver Advanced Analysis 2.0 negative binomial analysis, rather than direct summaries of individual sample data. Figure 5 illustrates the differential expression of genes compared to a naïve control at the acute 4-week time point. Within the panel of 146 genes related to neuroinflammation and oxidative stress, MARE probes have 62 unique differentially expressed genes, and NeuroNexus probes have 4 (Fig. 5a). Additionally, there are 25 genes differentially expressed in both probe types. In the volcano plots (Fig. 5b, c), the probe type is compared to a baseline of naïve control. A log_2_(fold change) >0 indicates upregulation from naïve control, and a log_2_(fold change) <0 indicates downregulation from naïve control. In addition to having a larger number of significantly differentially expressed genes at 4 weeks, the genes differentially expressed from the MARE group have a higher average log_2_(fold change) than NeuroNexus when compared to naïve control, suggesting a higher degree of upregulation for the significantly differentially expressed genes in the MARE probe groups. The enhanced number of transcripts related to the neuroinflammatory response observed around the MARE at the acute time point may be attributed to the larger cross-sectional area compared to the NeuroNexus probes, which results in more tissue displacement during insertion and a greater surface area for inflammatory cell accumulation. Given that gene expression was measured from homogenized tissue within 500 µm of the implant, the early response may be most strongly influenced by implant size rather than mechanical properties or therapeutic effects.

**Fig. 6:**
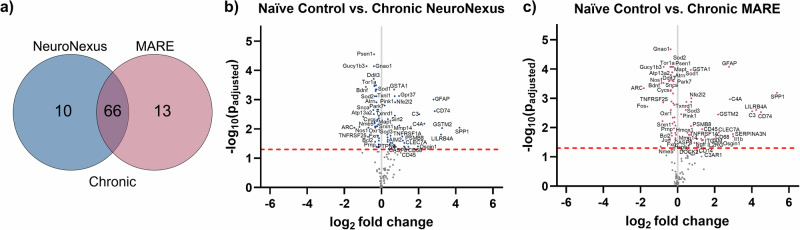
Differential expression for the NeuroNexus and MARE probes vs. naïve control at a chronic time point of 12 weeks. **a** Venn diagram showing the number of significant differentially expressed genes between the groups, not accounting for upregulation vs. downregulation. **b, c** Volcano plots showing each probe type, where each point is a gene in the panel. The dashed red line shows the significance threshold of *p*_adjusted_ < 0.05. Significant differentially expressed genes are labeled and shown in color. Some significant genes were not labeled due to space limitations. Genes with a log_2_ fold change >0 are upregulated from naïve control, and genes with a log_2_ fold change <0 are downregulated from naïve control.

The differential expression of genes at the chronic time point is illustrated by the corresponding Venn diagram and volcano plots in Fig. 6. At the chronic, 12-week time point, there are 66 common genes differentially expressed among both probe types relative to naïve controls, with 13 unique genes differentially expressed in the MARE group and 10 in the NeuroNexus group (Fig. 6a). The volcano plots for the chronic time point show similar levels of differential expression amongst the NeuroNexus and MARE groups in terms of numbers and average log_2_(fold change) compared to the acute time point (Fig. 6b, c). The results suggest that by the chronic time point, the strong response to the MARE implant has attenuated to more closely match the NeuroNexus response. Supplementary Fig. S4 shows the Venn diagrams and volcano plots for the MA group for both the acute and chronic time points.

**Fig. 7:**
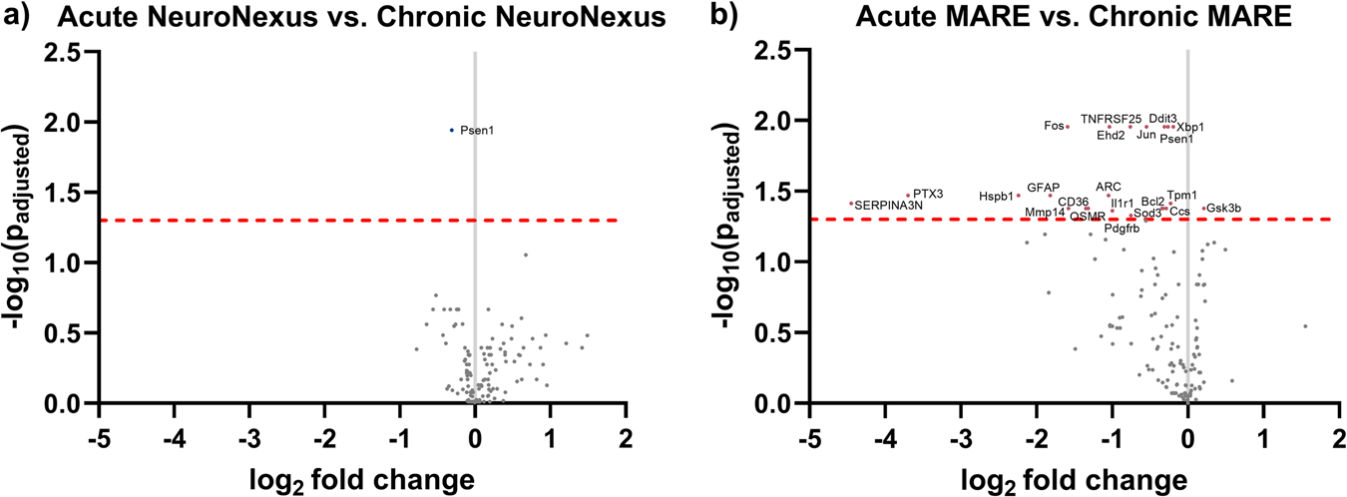
Differential expression from the acute (4 week) to chronic (12 week) time points for the NeuroNexus and MARE probes. **a** Volcano plot showing the differential expression for the chronic time point with a baseline of the acute time point for the NeuroNexus group. **b** Volcano plot showing the differential expression for the chronic time point with a baseline of the acute time point for the MARE group. Each point is a gene in the panel. The dashed red line shows the significance threshold of *p*_adjusted_ < 0.05. Significant differentially expressed genes are labeled and shown in color. Genes with a log_2_ fold change >0 are upregulated from naïve control, and genes with a log_2_ fold change <0 are downregulated from naïve control.

Additionally, the volcano plots showing differential expression of genes between time points for the NeuroNexus and MARE groups are in Fig. 7. The NeuroNexus group only had 1 significant differentially expressed gene from the acute to chronic time point, *Psen1*, while the rest of the analyzed genes demonstrated non-statistically significant differential expression levels (Fig. 7a). The MARE group had 21 significantly downregulated genes at the chronic time point relative to the acute time point, with 1 gene significantly upregulated, shown in Fig. 7b. The visualization of the levels of the differential gene expression supports that the NeuroNexus group experienced a stagnation of the healing tissue response at the chronic phase, while the MARE probes showed decreased expression of genes related to neuroinflammation and oxidative stress.

**Fig. 8:**
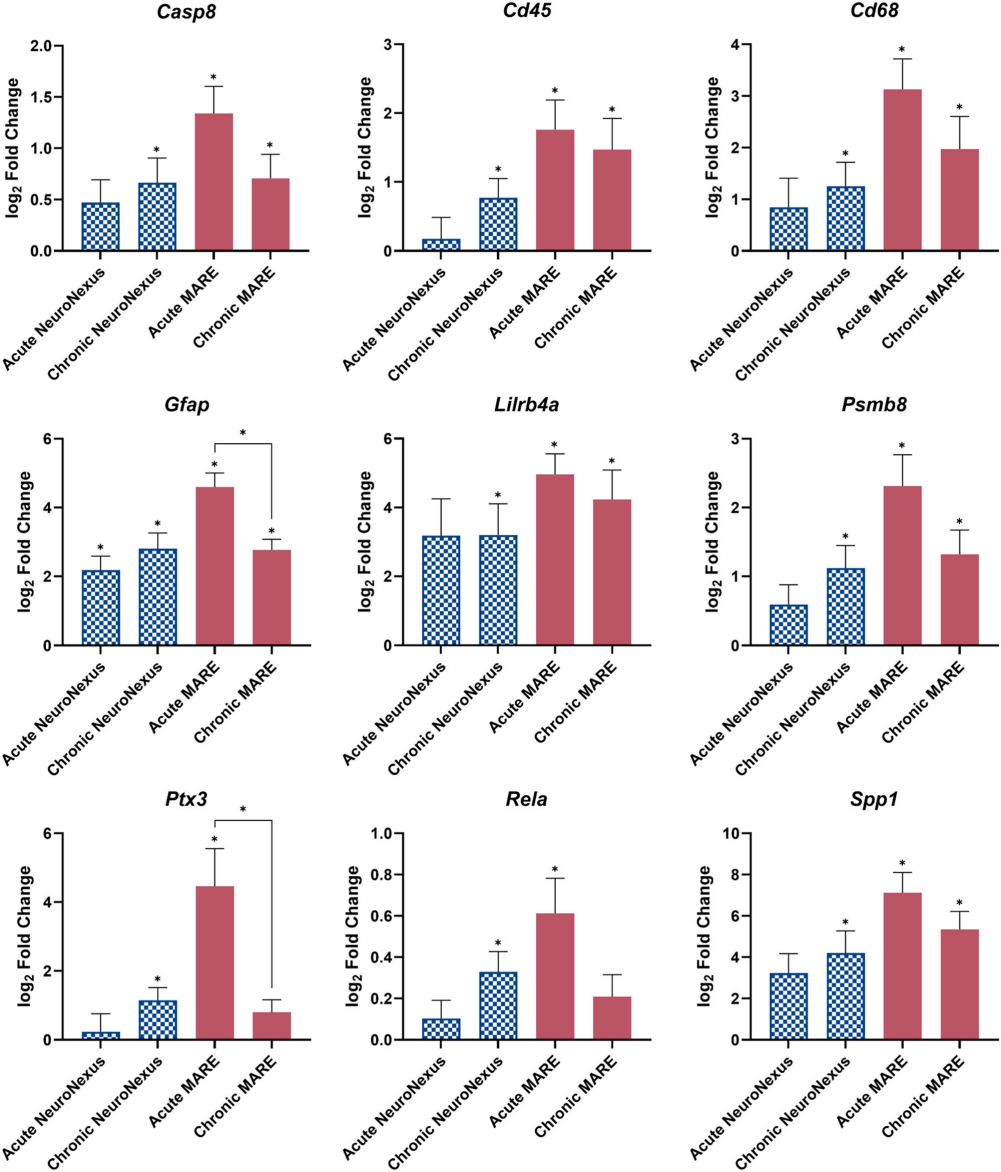
Selected genes related to neuroinflammation and neuronal function in terms of log_2_ fold change for the NeuroNexus and MARE probes at the acute and chronic time points. Error bars represent standard error. Statistical significance is denoted by * for *p* < 0.05.

Selected genes related to neuroinflammation and neuronal function in terms of log_2_(fold change) compared to naïve control are shown in Fig. 8. *Casp8*, a gene that plays a crucial role in apoptosis^88^, is not significantly expressed relative to naïve controls at the acute time point in the NeuroNexus group, but has a higher log_2_ fold change and is significant in the MARE group. At the chronic time point, *Casp8* expression increased in the NeuroNexus group, but decreased in the MARE group relative to the naïve control. *Cd45*, a marker for infiltrating leukocytes^89^, *Cd68*, a marker for activated microglia and macrophages^90^, *Lilrb4*a, a gene that modulates the inflammatory environment and microglial activation^91^, *Psmb8*, a gene that is involved in antigen presentation^92^, and *Spp1*, a gene that is involved in the activation and recruitment of immune cells such as microglia^93^, all follow the same trend as *Casp8*. *Rela*, which plays a role in the NF-κB pathway^94^, also exhibits that trend, though at the chronic time point in the MARE group is not significantly differentially expressed compared to naïve control. Finally, *Gfap*, a gene that is a marker for reactive astrocytes^95^, and *Ptx3*, a gene that plays a role in pattern recognition and the activation of complement pathways^96^, both exhibited a statistically significant decrease in differential expression from the acute to chronic phases in the MARE group. These specific genes, as well as the overall trends seen in the volcano plots, indicate that the NeuroNexus group has a stagnant or worsening neuroinflammatory response. In contrast, the MARE group shows improvement, even though there was a greater initial foreign body response.

**Fig. 9:**
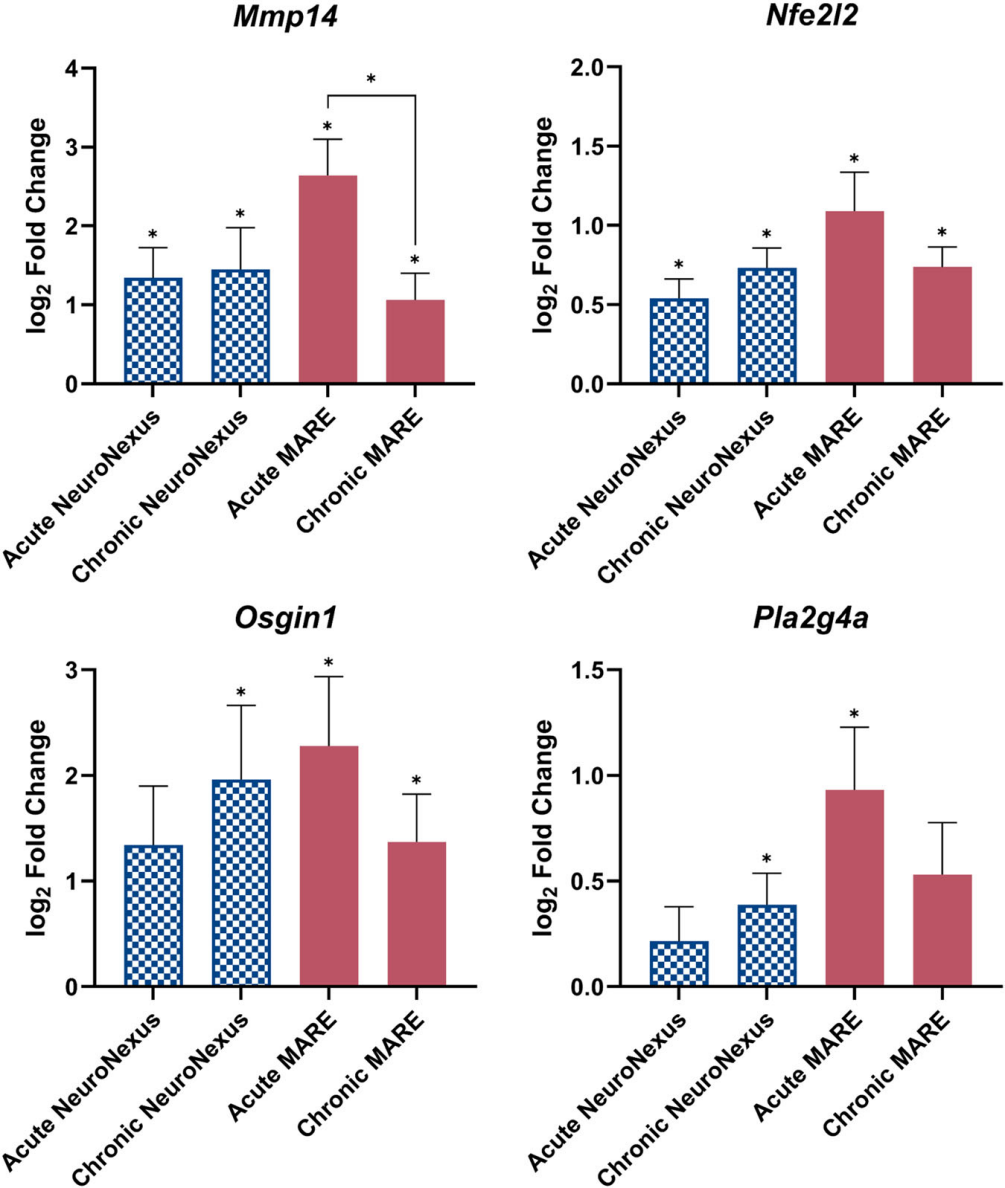
Selected genes related to oxidative stress in terms of log_2_ fold change for the NeuroNexus and MARE probes at the acute and chronic time points. Error bars represent standard error. Statistical significance is denoted by * for *p* < 0.05.

Selected genes related to oxidative stress are shown in Fig. 9. Like the genes related to neuroinflammation, *Osgin1*, a gene that regulates cell growth and survival under oxidative stress conditions^97^, has an increase in log_2_(fold change) from the acute to chronic time point in the NeuroNexus group, and a decrease in the MARE group. In addition, the level of differential expression, initially not significantly upregulated at the acute time point, became statistically significant at the chronic time point for the NeuroNexus group. *Pla2g4a*, a gene that influences lipid metabolism and inflammation^98^, exhibits a similar trend. *Nfe2l2*, a gene that influences the cellular response to oxidative stress elicited by inflammation or injury^99^, also displays the same trend, with the exception that the gene is significant in the acute NeuroNexus group. Finally, *Mmp14*, a gene that is overexpressed under oxidative stress, plays a role in the remodeling of the extracellular matrix and experiences a statistically significant decrease from the acute to chronic phase in the MARE group^100^. Together with the genes related to neuroinflammation, the oxidative stress genes show a healing tissue response occurring in the MARE group, as opposed to the stagnation or worsening in the NeuroNexus group. All the genes analyzed in this study are reported as upregulated, downregulated, or not significantly differentially expressed for all the volcano plot comparisons can be found in Supplementary Table S6.

### Correlation of recording performance with gene expression

Pearson correlation between the final week active electrode yield (AEY) for each implant and associated log_2_(fold change) of gene expression were examined within each probe type and phase to identify key mechanisms that may be contributing to implanted microelectrode recording failure. At the acute 4-week time point for the MARE group, there was a significant correlation (*p*_unadjusted_ <0.05, |r| > 0.75) between log_2_(fold change) of gene expression and AEY for only *Tnfrsf25*, which stimulates NF-κB and regulates cell apoptosis^101,102^. If expanding to all |r| > 0.75 (Supplementary Table S7), the genes with the strongest correlation to AEY are most closely associated with gene ontology (GO) terms related to neuronal apoptosis (GO:0043066—negative regulation of apoptotic process, GO:0006915—apoptotic process, GO:0043065—positive regulation of apoptotic process, and GO:0051402-neuron apoptotic process)^103,104^. At the acute 4-week end time point for the non-resveratrol releasing MA group, expression of 13 genes was significantly correlated with final week AEY (Supplementary Table S7). The acute MA group significantly correlated genes are most closely associated with GO:0006979—response to oxidative stress and KEGG pathway mmu05022: Pathways of neurodegeneration. At the acute 4-week time point for the NeuroNexus group, there was a significant correlation between log_2_(fold change) of gene expression and AEY for 13 genes, listed in Supplementary Table S7. The GO terms most closely associated with the significantly correlated genes for the acute NeuroNexus group are GO:0006979—response to oxidative stress, GO:0043525—positive regulation of neuron apoptotic process, and GO:1900272—negative regulation of long-term synaptic potentiation. These findings indicate that the biological processes underlying recording performance differ across the probe types at the acute time point. The strong correlation between genes related to oxidative stress and active electrode yield observed for the acute MA and NeuroNexus groups, but not the MARE group, suggests that the antioxidative effects of resveratrol reduce the contributions of oxidative stress to recording performance. Additionally, in contrast to the MA group, the absence of correlation with pathways of neurodegeneration in the MARE group implies that resveratrol may also mitigate the impact of neurodegeneration on neural recording metrics. Instead, in MARE probes in the acute phase, recording performance is more closely linked to neuronal apoptosis.

At the chronic 12-week time point for the MARE group, there was a significant correlation between log_2_(fold change) of gene expression and AEY for 5 genes: *Atp13a2*, which is related to neurite outgrowth^105^; *Mapt*, which promotes neuron microtubule assembly and function^106^; *SPP1*, which encodes for a pro-inflammatory cytokine associated with activated microglia^107^; *Tor1a*, which is protective against oxidative stress^108^; and *Nr2f6*, a transcriptional repressor of cytokines^109^. These genes are most closely associated with GO:0043005—neuron projection, GO:0042995—cell projection. At the chronic 12-week time point for the MA group, expression of 7 genes were significantly correlated with AEY. The significantly correlated MA genes are most closely associated with KEGG pathway mmu04066—HIF-1 signaling pathway, which may be induced by nitric oxide or low oxygen availability^110^. At the chronic 12-week time point for the NeuroNexus group, there was significant correlation between log_2_(fold change) of gene expression and AEY for 11 genes. The most closely associated GO terms for the significantly correlated genes are GO:0030593—neutrophil chemotaxis, GO:0006954—inflammatory response, GO:0090026—positive regulation of monocyte chemotaxis, GO:0001774—microglial cell activation, and GO:0071347—cellular response to interleukin-1). There was no overlap in significantly correlated genes between MARE and NeuroNexus or between MARE and MA groups at either end time point. At the 4-week time point, both MA and NeuroNexus AEY was significantly correlated with expression of 4 genes (Supplementary Table S7): *Mutyh* is involved in repairing oxidative DNA damage^111^, *Prnp* encodes a prion protein involved in defense against oxidative stress^112^, *Tor1a, and Abl1* is activated by oxidative stress and influences cellular fate^113^. Together, these findings suggest that in the chronic phase, active electrode yield is most strongly affected by different processes for each probe type. In the MARE group, correlations were linked to genes associated with neuronal structure and repair, with minimal involvement of neuroinflammatory or oxidative stress pathways. Resveratrol eluted early after implantation may have lasting impacts on the surrounding tissue even after depletion from the probe. In contrast, the MA and NeuroNexus groups showed stronger associations with hypoxic signaling and the innate immune response, respectively, indicating that unresolved oxidative stress and inflammation continue to affect recording outcomes in these groups. The lack of overlap in correlated genes between MARE and the other probe types is indicative of the distinct tissue response elicited by the MARE platform.

**Fig. 10:**
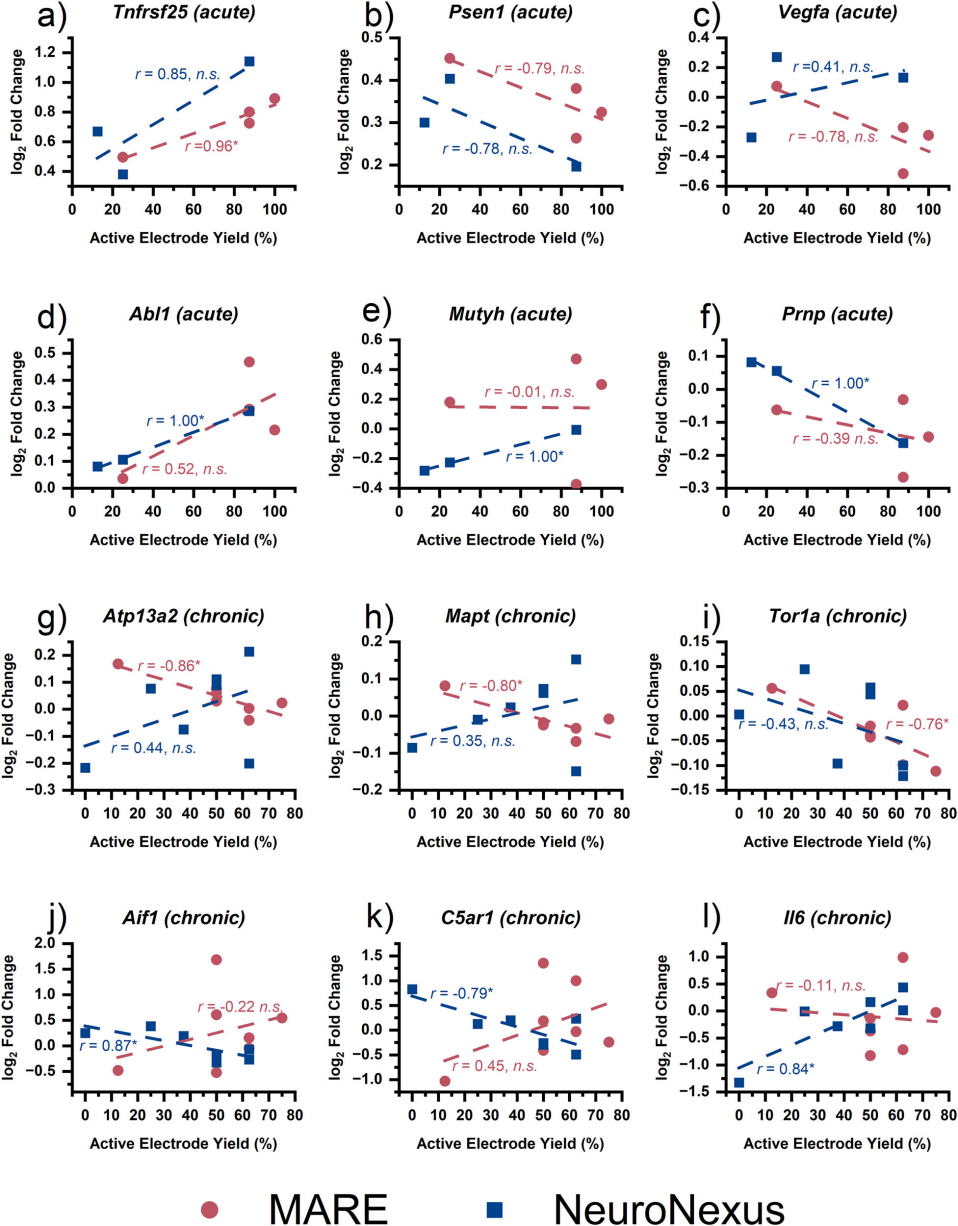
Relationship between active electrode yield and gene expression. Correlation between active electrode yield and selected genes related to apoptotic processes (**a**–**c**), oxidative stress (**d**–**f**), neuronal projection (**g**–**i**), and inflammation response (**j**–**l**) for the NeuroNexus and MARE probes at the acute (**a**–**f**) and chronic time points (**g**–**l**). Each point represents one implant, with one implant per animal. Statistical significance is denoted by * for *p* < 0.05.

The relationship between the expression of selected genes and AEY is shown in Fig. 10. Gene expression and probe recording performance at the acute time point are shown in Fig. 10a–f and for the chronic time point are shown in Fig. 10g–l. Select genes associated with GO:0006915—apoptotic process are shown in Fig. 10a–c to highlight genes with relatively strong correlation to MARE recording at the acute time point. Both acute MARE and NeuroNexus groups have a positive correlation between AEY and log_2_(fold change) of *Tnfrsf25* (Fig. 10a), with the statistically significant correlation for the MARE group. *Psen1* (Fig. 10b) is involved in Amyloid-β precursor protein processing and has antioxidative functions^114^, and trends toward a negative correlation with AEY in both NeuroNexus and MARE groups. *Vegfa* (Fig. 10c) promotes neuronal migration, neuronal survival, and axon guidance^115^, and is negatively correlated with the acute MARE probe performance and positively correlated with the acute NeuroNexus probe performance. Select genes associated with GO:0006979—response to oxidative stress are shown in Fig. 10d–f to highlight genes with strong correlation to NeuroNexus recording at the acute time point. *Abl1* (Fig. 10d) showed positive correlation with AEY for both acute groups, with significant correlation with the acute NeuroNexus group. *Abl1* is activated under oxidative stress conditions^116^, *Mutyh* (Fig. 10e) is significantly positively correlated with acute NeuroNexus AEY, and not correlated with acute MARE AEY. *Prnp* (Fig. 10f) is significantly negatively correlated with AEY for the acute NeuroNexus group AEY and trends toward negative correlation with the acute MARE group AEY. Select genes associated with GO:0043005—neuron projection are shown in Fig. 10g–i to highlight genes with relatively strong correlation to MARE recording at the chronic time point. *Apt13a2* (Fig. 10g), *Mapt* (Fig. 10h), and Tor1a (Fig. 10i) are all significantly negatively correlated with chronic MARE group AEY. *Atp13a2* and *Mapt* trend toward a positive correlation with AEY for the chronic NeuroNexus group, while *Tor1a* trends toward a negative correlation with chronic NeuroNexus group AEY. Select genes associated with GO:0006954—inflammatory response are shown in Fig. 10j–l to highlight genes with strong correlation to NeuroNexus recording at the chronic time point. *Aif1* (Fig. 10j) is a key intracellular protein that binds calcium and is recognized as a marker for the activation of microglia and macrophages within the central nervous system^117^, and is significantly negatively correlated with chronic NeuroNexus AEY and weakly positively correlated with chronic MARE AEY. *C5ar1* (Fig. 10k) is a receptor for the complement system and is significantly negatively correlated with chronic NeuroNexus AEY and weakly positively correlated with chronic MARE AEY. *Il6* (Fig. 10l) encodes a cytokine involved in modulating glial cell activity and is strongly positively correlated with chronic NeuroNexus AEY and not correlated with chronic MARE AEY. Together, the distinct gene expression-AEY correlation patterns indicate that the biological processes affecting recording performance differ between probe types. The MARE probe performance is more closely associated with neuronal health and apoptosis-related pathways, particularly at the chronic time point. In contrast, NeuroNexus recording performance is more closely related to ongoing inflammatory and oxidative stress responses.

**Fig. 11:**
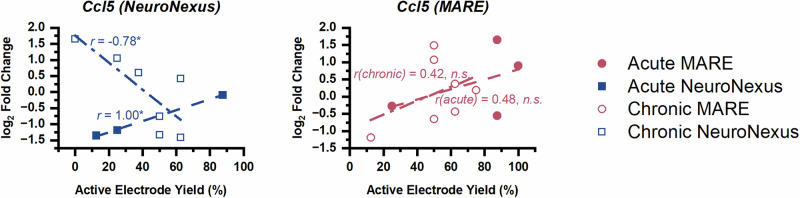
Correlation between active electrode yield and *Ccl5* for both acute and chronic NeuroNexus groups (left) and acute and chronic MARE groups (right). Each point represents one implant, with one implant per animal. Statistical significance is denoted by * for *p* < 0.05.

There were no genes that expressed a significant correlation with MARE AEY at both acute and chronic time points, even if the acute time point threshold is expanded to include all correlations with |r | > 0.75. In the NeuroNexus group, *Ccl5*, which encodes a neuroprotective chemokine, is significantly correlated with AEY at both the acute and chronic time points (Fig. 11). However, the direction of the correlation shifted from a positive correlation at the acute time point to a negative correlation at the chronic time point. In contrast, the MARE groups at both time points showed a moderate trend toward positive correlation with very similar slopes. After injury, the chemokine CCL5 reduces oxidative stress, aids in neuronal ATP generation, and promotes axon and synapse regeneration^118^. However, CCL5 has also been shown to have pro-inflammatory effects on microglia, and its overexpression has been associated with memory impairment in mice^119,120^. Therefore, the role of *Ccl5* may initially be aiding in neuroprotection and regeneration in the NeuroNexus groups, but later becomes more indicative of an inflammatory state associated with poor recording function.

## Discussion

The current study establishes the MARE probes as a platform technology capable of chronic intracortical recording of single units. Even with larger cross-sectional and surface areas, the MARE matches or outperforms the standard silicon-based NeuroNexus probes for recording metrics during each implant phase. Further, MARE probes exhibited more stable 1 kHz impedance magnitudes over time compared to the NeuroNexus probes, indicating improved device and tissue stability. Together, the active electrode yield, units per channel, and impedance results support the central hypothesis that MARE probes will better integrate with tissue and improve neural recording performance compared to rigid silicon-based probes. The combination of the flexibility of the mechanically-adaptive substrate and the antioxidant elution stabilized and promoted healing at the MARE probe-tissue interface.

MARE probes showed steady active electrode yield and units per channel over the acute and subchronic phases, followed by a decline at the chronic time point. The timing of the onset of performance deterioration aligns with the depletion of resveratrol, and therefore the exhaustion of its antioxidative therapeutic effects^68^. After resveratrol depletion, the general decrease in active electrode yield follows trends from other chronic recording studies. Long-term resveratrol release from the MARE probes may prolong the improvements in recording performance.

It is important to note that the MARE and NeuroNexus probes differ in both electrode site size and material, which introduces tradeoffs in expected signal quality. The larger 30 µm-diameter gold electrodes on the MARE probes are expected to exhibit lower signal amplitudes due to spatial averaging, as well as greater biological noise from a larger recording volume^121^. In contrast, the 15 µm-diameter iridium electrodes on the NeuroNexus probes provide improved spatial resolution and improved spike discrimination and are therefore generally preferred for isolating single-unit activity. However, larger recording sites, such as those on the MARE probes, have a lower thermal noise contribution due to their lower impedance. Despite being potentially disadvantaged in a comparison, MARE probes matched or exceeded the recording performance of NeuroNexus probes at subchronic and chronic time points. The improved tissue integration facilitated by mechanical compliance and transient resveratrol delivery from the MARE probes may facilitate the presence of healthy, active neurons close to the recording sites.

The repolarization rate analysis shows that MARE probes recorded more units/electrode site from fast-repolarizing, putative inhibitory neurons throughout the study. As putative inhibitory neurons are more susceptible to damage from oxidative stress and trauma^122^, it is notable that MARE probes protected inhibitory neurons in comparison to NeuroNexus probes. Studies of traumatic brain injury and schizophrenia disease models indicate that oxidative stress is negatively correlated with the integrity of inhibitory neurons, affecting neural circuits^123–125^. Reduction in the balance between inhibitory and excitatory activity can result in unstable chronic recording quality^85^. As signals recorded from both MARE and MA probes demonstrated similar trends, mechanical compliance appears to affect the local neuronal populations more than resveratrol delivery. However, the MARE probes recorded more fast-repolarizing units/electrodes in the subchronic phase, similar to the AEY and units per channel trends, indicating that the resveratrol delivery may also play a role in inhibitory neuron protection. The flexible, polymer-based NC substrate with viscoelastic properties may provide selective frequency-damping capabilities that can allow detection of physiological signals, such as neuronal single units, with a high signal-to-noise ratio and low mechanical noise^126^. Such filtering may allow the NC-based probes to successfully record from more putative inhibitory neurons rather than surrounding excitatory neurons. Additionally, the flexible, compliant substrate of MARE probes may promote improved device-tissue integration and reduced glial scarring around the device, contributing to the stable fast-repolarization rates across the acute, subchronic, and chronic phases. The synergistic environment may also preserve inhibitory neuronal health, leading to increased populations near the implant site compared to the stiff NeuroNexus devices. Additionally, the stiffness of the NeuroNexus probes may result in increased firing frequency of excitatory neurons, contributing to the increased recording rate for regular-repolarizing units/electrode sites. As the glial scar continues to form through the chronic time point, it causes neuronal death and dieback^1^, leading to the trend of recording fewer units/electrodes over time for both regular- and fast-repolarizing neurons.

Bulk gene expression analyses of 146 selected neuroinflammation and oxidative stress genes were expected to elucidate the local tissue state within 500 µm of the implanted probes. At the acute 4-week time point, MARE probes caused more genes related to oxidative stress and neuroinflammation to be differentially expressed compared to NeuroNexus probes. Further, many of the genes had a higher log2(fold change) in the MARE group than the NeuroNexus group at the acute time point. One reason for these observations is that MARE probes are significantly thicker than NeuroNexus probes, inducing more damage to neurons and glial cells upon implantation. Limitations in probe mechanics for insertion and electrode fabrication processes require the MARE probes to be larger. At just 4 weeks post-implantation, the benefits of mechanical compliance and local resveratrol delivery may not balance the exacerbated iatrogenic injury from the larger probe size. The larger probe size may also contribute to delayed wound healing due to increased vascular injury and blood-brain barrier compromise. Due to the nature of bulk gene analysis, the entire 1.6 µL volume of tissue is processed as one homogenous sample, and normalization for size and spatial characterization is not possible. The samples isolated with the 1 mm biopsy punch are comprised of the tissue surrounding the probe and a void corresponding to where the probe had resided within the tissue. Approximately 98.7% of the MARE and MA group sample volume is tissue, rather than the void produced by the implanted probe. In the NeuroNexus groups, approximately 99.7% of the isolated sample volume is tissue, rather than the void from the implanted probe. Therefore, the difference in tissue volume between the two sample types is only approximately 1%, which is well within typical sample measurement variability. Further, gene count normalization using housekeeping genes accounts for variability in measurement due to the tissue volume within the biopsy punched samples. Analysis of the homogenized sample does not indicate the local density of gene expression markers or glial scar thickness. Spatially-resolved immunohistochemistry analyses have shown elevated neuroinflammatory marker expression within 50 μm of the implant site^127^. Therefore, the ~60% larger MARE surface area may result in larger mRNA measurements compared to the thinner NeuroNexus probe.

By the chronic 12-week time point, there is a considerable increase in MARE and NeuroNexus group overlap in differentially expressed genes in comparison to naïve controls (Fig. 6). At the chronic time point, gene expression levels were similar between the NeuroNexus and MARE groups due to increased expression in the NeuroNexus group and decreased expression in the MARE group compared to the acute time point (Figs. 8 and 9). While decreased differential gene expression indicated healing and recovery for MARE and MA probes, the NeuroNexus group’s state of neuroinflammation and oxidative stress remained stagnant or grew slightly worse. Additionally, when looking at specific pathways related to genes, the acute MARE vs. naïve control had altered toll-like receptor signaling and ECM-receptor interaction pathways, through the upregulation of genes such as *Spp1*, *Irf7*, *Cd14*, *Casp8*, *Rela*, *Il1b*, *Cd36*, and *Fn1*. Both pathways became insignificant at the chronic time point, indicating a return to baseline of inflammation compared to naïve control. However, the chronic NeuroNexus group had an altered neurodegeneration gene pathway, indicating that genes contributing to neurodegeneration were affected at a chronic time point in the NeuroNexus group compared to naïve control. Twenty-two total genes were differentially expressed in the neurodegeneration pathway, with 4 upregulated and 18 downregulated. Because the acute and chronic MARE groups did not display significantly altered neurodegeneration pathways, this finding could indicate that NeuroNexus probes may demonstrate increased neurodegeneration at a chronic time point. Individual genes may be pro- or anti-inflammatory; however, differential gene expression in comparison to naïve control indicates that the tissue environment is altered in response to the implanted probe. The amelioration of neuroinflammation and oxidative stress can indicate good integration of the probe into cortical tissue and is consistent with the improvement in active electrode yield and units per channel in MARE probes after the acute phase. Our interpretations of the gene expression results are further supported by immunohistochemistry (IHC) from representative NeuroNexus and MARE samples at the 12-week time point (Fig. S5). Notably, the IHC images indicate the presence of neurons very near to the MARE implant site, in contrast with a larger gap between the NeuroNexus implant site and neurons.

In seeking to identify the key mechanisms influencing the ability to detect units from each probe type, we examined the active electrode yield during the final week for each probe and the log_2_(fold change) of the expression of each gene for significant correlation. The gene expression-active electrode yield plots in Figs. 10 and 11 highlight the variability in performance and gene expression across probe types, which may be explained by the variation in vascular damage during the implant process. In some cases (e.g., *Psen1, Atp13a2 and Mapt* for MARE probes and *Mutyh, C5ar1, and Il6* for NeuroNexus probes), a trend toward naïve control levels was associated with improvements in recording quality. However, in other cases (e.g., *Tnfrsf25, Vegfa, Abl1*, and *Tor1a*), a trend toward overexpression or underexpression is associated with improvements in active electrode yield, underscoring the complexity of pathways associated with neuroinflammation, oxidative stress, and neuronal health. *Ccl5*, for example, is significantly correlated with AEY during both the acute and chronic end time points, though the correlation switches from a positive correlation at 4 weeks to a negative correlation at 12 weeks. This shift in correlation over time emphasizes the dynamic nature of gene regulation and its multifaceted impact on probe performance, suggesting that temporal changes in gene expression play a critical role in the long-term stability and functionality of neural recording interfaces. Recording with the MARE devices was most strongly correlated with neuronal health (acute) and structure (chronic), while recording with the MA devices was most strongly correlated with responses to high concentrations of reactive oxygen species and reactive nitrogen species. At the acute end time point, NeuroNexus recording was most strongly correlated with both oxidative stress response and neuron apoptosis regulation. At the chronic end time point, NeuroNexus recording was most strongly associated with indicators of an inflammatory response. Consistent with findings from Whitsitt et al., genes that serve as cell-type specific markers associated with astrogliosis (e.g., *Gfap* and *Cd68*) were generally less strongly correlated with active electrode yield than genes more strongly associated with oxidative stress and neuroinflammatory processes, though *Cd68* was significantly correlated with NeuroNexus AEY at the acute end time point. The lack of a strong correlation between genetic markers of neuroinflammation and recording for MA and MARE probes suggests that mechanical compliance may have sufficiently mitigated the effects of neuroinflammation on recording. Further, the lack of a strong correlation between genetic markers of oxidative stress and recording for MARE probes suggests that resveratrol release may sufficiently mitigate oxidative stress as an influence on recording performance. Our results further suggest that recording performance of rigid silicon-based probes like the NeuroNexus probes may be improved by mitigating oxidative stress at early time points, followed by addressing neuroinflammation at later time points. In contrast, MARE probe performance may be enhanced with local delivery of agents directed toward promoting neuronal health. It is important to note that there may be delays between changes in gene expression and related changes in active electrode yield, and that stronger or an increased number of correlations may arise if AEY could be measured in the days or weeks after gene expression measurements.

Overall, the heightened inflammatory gene expression in the acute time point followed by a reduction into the chronic phase (Fig. 7) indicates healing and improvement in the tissue environment. The similar or improved AEY and units per channel for MARE probes compared to NeuroNexus probes in the absence of corresponding similar or improved gene expression profiles may be explained by the bulk gene expression measurement within a volume 500 µm from the probe. Given that the recording microelectrodes are on only one surface of the probe, the additional interfacial inflammation promoted by increased probe surface area may not impact recording. Spatial transcriptomics would be needed to understand the relative gene expression density within the critical 50–100 µm of the recording microelectrodes. Further, genes not included in the 146 gene panel for oxidative stress and neuroinflammation may be needed to more completely explain the tissue environment. Mechanisms underlying the recording performance were found to depend on the probe type, with MARE probe performance most affected by neuronal health, MA probe performance most affected by oxidative stress, and NeuroNexus probes initially most affected by oxidative stress and later by neuroinflammation. Together, our findings underscore the role of implant mechanical behavior and local antioxidant delivery in the tissue environment, establishing MARE probes as a mechanically adaptive, therapeutic-eluting platform that supports chronic neural recording performance.

## Methods

### Material dispersion and film casting

The polymer-based nanocomposite (NC) substrate for the mechanically-adaptive, resveratrol-eluting (MARE) and mechanically-adaptive (MA) neural probes was prepared using a film casting, evaporation, and planarization method as detailed in previous publications^27,63,64,66,67,79–81,128–132^. Briefly, dispersions of tunicate cellulose nanocrystals (Cellulose Lab, Fredericton, New Brunswick, Canada), polyvinyl acetate (Sigma-Aldrich, St. Louis, MO, USA), and trans-resveratrol (Mega Resveratrol, Danbury, CT, USA) in dimethylformamide (68-12-2, Fisher-Scientific, Hampton, New Hampshire, USA) were prepared separately and then combined. The relative amounts of polyvinyl acetate, cellulose nanocrystals, and trans-resveratrol were chosen to synthesize films with 15% w/w cellulose nanocrystals and 0.01% trans-resveratrol in the polyvinyl acetate matrix^68^. The homogenous mixture was then cast on a layer of Sylgard 184 silicone elastomer (Dow Chemical Company, Midland, Michigan, USA) in a low-form PTFE evaporating dish (Fisher-Scientific, Hampton, New Hampshire, USA). The dish was then placed in a vacuum oven (Isotemp Vacuum Oven Model 280 A, Fisher Scientific, Waltham, MA, USA) at 70 °C for approximately 1 week to evaporate the dimethylformamide. Once fully dry, the film was removed from the dish and stored in a dry box. For further reduction of surface roughness on the film, the film was pressed between a set of PDMS-coated acrylic discs in a Carver laboratory press for 15 min at a temperature of 90 °C and a pressure of 3000 psi. After cooling, films were separated from the disc, and the surface roughness was measured at 3 different places with a Dektak stylus profilometer (Bruker, Billerica, MA, USA). Films with a surface roughness measurement average of less than 500 nm were considered suitable for photolithographic patterning.

### Electrode fabrication and packaging

Once the films were prepared and pressed, functional recording electrodes were fabricated on the NC substrates as described in previous work^79,80^. A 2 μm-thick Parylene C barrier layer was vapor-deposited onto both sides of the substrate using an SCS PDS 2010 Parylene Coater (Specialty Coating Systems, Indianapolis, IN, USA). The Parylene C-coated film was then mounted to a carrier wafer using Kapton tape around the edges of the film. A 20-nm thick titanium adhesion layer followed by a 200 nm-thick gold conductive layer was then sputter-deposited onto the wafer using a Denton Vacuum Explorer 14 deposition system (Denton Vacuum, Moorestown, NJ, USA). AZ P4330 photoresist (EMD Performance Materials, Darmstadt, Germany) was spin-cast onto the metal layer, followed by a soft bake for 60 s at 110 °C, and then i-line UV light exposure with an exposure dose of 104 mJ/cm^2^ in a photomask aligner (Karl Suss MA6, Suss Microtec, Garching, Germany). Next, the photoresist was developed by immersion in AZ 400 K 1:4 developer (EMD Performance Materials, Darmstadt, Germany) for 2 min and then rinsed with deionized water. With the photoresist mask, the Au layer was wet-etched using Au etchant TFA (Transene Company Inc., Danvers, MA, USA) for 50 s, and the Ti layer was wet-etched using buffered oxide etchant (Transene Company Inc., Danvers, MA, USA) for 20 s. Following metal patterning, a second 2 μm-thick Parylene C insulation layer was vapor-deposited onto the substrate. The Parylene C layer was patterned by spin-casting AZ nLOF 2070 photoresist (EMD Performance Materials, Darmstadt, Germany) onto the wafer, followed by a soft bake at 110 °C for 90 s. With an exposure dose of 176 mJ/cm^2^, the wafer was exposed in a mask aligner, then post-exposure baked on a hot plate at 110 °C for 90 s. The photoresist was then immersion-developed for 2 min in 300 MIF developer (EMD Performance Materials, Darmstadt, Germany) and rinsed with deionized water. Following development, the Parylene C was then dry-etched with oxygen plasma with a MARCH CS 1701 plasma tool in an environment of 85% O_2_ and 15% CF_4_ at a power of 200 W to expose the connector and recording contacts. After Parylene C etching, individual probes were laser-micromachined with a picosecond laser (Oxford Lasers, Oxfordshire, UK) and released from the carrier wafer. The individual probes were then functionally connected to a device package using a conductive epoxy (8330-19G, MG Chemicals, Burlington, Ontario, Canada) to interface with electronics for neural recording and impedance measurements^79^. The package consists of a custom-made printed circuit board (Advanced Circuits, Aurora, CO, USA), with connectors (DF30FC-20DS-0.4V(82), Hirose, Downers Grove, IL, USA) as well as ground and reference wires soldered onto their respective contact pads. Insulating epoxy (EP21LVMed, Master Bond, Hackensack, New Jersey, USA) was applied over all electrically active components on the PCB to isolate the system.

### Electrochemical impedance spectroscopy

Electrodes on the MARE, NeuroNexus, and MA probes were tested with electrochemical impedance spectroscopy prior to implantation and throughout the in vivo study, as described previously^79^. A 3-electrode configuration was used for both benchtop and in vivo testing using a BioLogic SP-300 potentiostat (BioLogic, Seyssinet-Pariset, France). The experimental parameters consisted of a 10 mV amplitude with a frequency sweep from 1 Hz to 100 kHz. Benchtop EIS testing in a 1X PBS solution was conducted on all probes to generate a baseline impedance measurement and ensure that the electrodes were suitable for extracellular neural recording once implanted in vivo. Each probe was generally considered acceptable for neural recording and therefore, in vivo implantation if the magnitude of most channels at 1 kHz were between 100–1000 kΩ^14,38,133–135^.

### In vivo implantation surgeries

All animal procedures were done in accordance with established protocols approved by the Institutional Animal Care and Use Committee at the Louis Stokes Cleveland VA Medical Center. Male Sprague-Dawley rats weighing 250–300 g were implanted with a MARE probe, silicon NeuroNexus neural probe (A1x16-3mm-100-177-Z16, NeuroNexus, Ann Arbor, MI, USA), or MA probe for a duration of 4 (*n* = 5 for MARE, *n* = 4 for NeuroNexus and MA) or 12 weeks (*n* = 7 for each probe type). One animal died prematurely from the acute NeuroNexus and MA groups, and was therefore not included in the study. MARE and MA probes had 8 recording channels (Fig. 1), and NeuroNexus probes had 16 recording channels, with equal spacing of 100 μm center-to-center for the recording sites on both types of devices. To control for depth, only the bottom 8 channels of the NeuroNexus probes were considered in the recording, repolarization rate, impedance, and recording-gene expression correlation analyses.

The rat was anesthetized with 2.5% isoflurane gas for knockdown and throughout the procedure. Clippers were used to shave the surgical area on the head and lubricant was applied to the eyes. Before surgery, each rat was administered a dose of the local analgesic Marcaine (0.15 mL of 0.25%), the antibiotic cefazolin (16 mg/kg), and the analgesic meloxicam (1 mg/kg) subcutaneously. After drug administration, the animal was moved onto a stereotaxic frame to fix the head during surgery. The surgical site was prepared with betadine and isopropanol. The rat’s vitals were monitored throughout the procedure with a MouseSTAT Pulse Oximeter and Heart Rate Monitor (Kent Scientific Corp., Torrington, CT, USA). An incision was made on the top of the head to expose the skull. The periosteum was removed, followed by the application of hydrogen peroxide and VetBond (3 M, St. Paul, MN, USA) to dehydrate and condition the skull for drilling. Craniotomies for the reference (1.5 mm lateral, 5.5 mm posterior to bregma) and ground (1.5 mm lateral, 1.5 mm posterior to bregma) bone screws were drilled with a 1.35 mm drill bit and Kopf dental drill (David Kopf Instruments, Tujunga, CA, USA). The craniotomy for the neural probe (3 mm lateral, 2 mm posterior to bregma on the contralateral side as bone screws) was drilled with a 1.75 mm drill bit. The drill was pulsed, and saline was applied to minimize thermal heating and consequential inflammation^136,137^. The bone screws were positioned with a screwdriver (Stoelting, Wood Dale, IL, USA). The neural probe was attached to the stereotaxic arm using a ZIF clip headstage and slowly lowered over the craniotomy. Ground and reference wires were wrapped around the bone screws and secured with dental cement (A-M Systems, Sequim, WA, USA) prior to probe implantation. The electrode was then implanted 2 mm into the cortex. The craniotomy was sealed with Kwik-Cast (World Precision Instruments, Sarasota, FL, USA), and dental cement was layered to form a headcap. Sutures were placed to close the wound while allowing access to the protruding connector on the neural probe. To manage post-operative pain, a dose of meloxicam (1 mg/kg) was given the following 2 days after surgery. Two doses of cefazolin (16 mg/kg) were administered the day after surgery.

### In vivo EIS testing

In vivo electrochemical impedance spectroscopy was performed weekly to characterize electrode impedance over time and correlate the findings with neural recording performance. Animals were anesthetized with 2.5% isoflurane gas throughout in vivo impedance testing. The exposed connector was scrubbed with isopropanol to remove any debris. The reference lead was electrically connected to the ground bone screw, the counter lead to the reference bone screw, and the working lead to the individual recording electrodes on the neural probe via a breakout board. Any electrode with 1 kHz impedance >2.5 MΩ or <100 kΩ was considered non-viable and excluded from the analysis. The geometric mean and geometric standard deviation factor of the impedance magnitude were calculated for each week and probe type over the 12-week period. A Shapiro–Wilk test indicated the data followed a normal and lognormal distribution. A two-way ANOVA tested for significance between probe types and time points. Statistical significance is denoted by **** for *p* < 0.0001.

### In vivo neural recording and analysis

In vivo neural recording sessions were completed on the day of surgery and biweekly for the duration of the study to track and evaluate recording performance over time. Following established protocols for single-unit neural recording^79,82,138,139^, animals were anesthetized using 2.5% isoflurane gas from the previous in vivo EIS session. The animal was then placed in a custom acrylic recording chamber. In the chamber, a ZC-16 ZIF-clip headstage was clipped into the exposed connector. The headstage was connected to an ACO32 Commutator that allowed the animal to move while minimizing motion artifacts and then to a RA16PA 16 Channel Medusa Pre-Amp to sample the signal at 24414.1 Hz. The digitized signal from all channels was acquired using the RZ5 Bioamp Processor (Tucker-Davis Technologies, Alachua, FL, USA) using Synapse software. The 10-min recording session started once the animal was awake and was returned to its home cage when finished.

Single unit recording activity was analyzed using Plexon Offline Sorter (Plexon Inc., Dallas, TX, USA). Common median referencing was applied to remove noise and common mode signals. Single units were identified with a −4σ standard deviation threshold from the mean. Additionally, high-amplitude (±500 μV) and cross-channel artifacts were invalidated. An automated K-means scan was used to sort spikes, and verification was completed manually. Further analysis of recording metrics such as peak-to-peak amplitude and signal-to-noise ratio was performed using MATLAB (Mathworks, Natick, MA, USA). Peak-to-peak amplitude was calculated as the voltage range from the peak to the valley of each unit. The signal-to-noise ratio was calculated using a proportion of the peak-to-peak amplitude to the noise for each unit.

Recording data was sorted into three groups based on phases correlated to the neuroinflammatory response. The acute phase was defined as weeks 1–5, the subchronic phase as weeks 6–11, and the chronic phase as week 12 in this study^10,76,140^. For each phase, active electrode yield was calculated as a proportion of the active electrodes to the total number of electrodes. Units per channel were calculated as a proportion of the units recorded to the total number of electrodes. For 4-week implant groups, 5 channels on NeuroNexus probes, 6 on MA probes, and 3 on MARE probes were excluded from analysis as they did not record a single unit action potential during any recording session. For the 12-week implant groups, 1 channel on NeuroNexus probes and 0 channels on both MA and MARE probes were excluded from the analysis. For statistical analysis, a two-tailed proportion *z*-test was run in R Studio (RStudio, PBC, Boston, MA, USA) to compare active electrode yield and units per channel across the MA, MARE, and NeuroNexus groups and acute, subchronic, and chronic phases. Statistical comparison of peak-to-peak amplitude and signal-to-noise ratio across groups and phases were completed in GraphPad Prism (Dotmatics, Boston, MA, USA). The data had a non-parametric distribution, so a Kruskal–Wallis test and a Benjamini–Krieger–Yekutieli procedure were used for multiple comparisons to reduce type I error and increase power.

### Repolarization rate analysis

The repolarization rate classification for each single unit action potential waveform was used to determine the relative contributions of fast-repolarizing, putative inhibitory neurons and regular-repolarizing, putative excitatory neurons to the recorded signal^85–87^. Carron et al. reported that putative inhibitory neurons are more susceptible to oxidative stress and trauma-induced damage than excitatory neurons^122^. Additionally, in many neurological disorders, such as stroke and traumatic brain injury, impairment or loss of inhibitory neurons may contribute to the imbalance of excitation and inhibition observed in the disease state^123,141,142^. As inhibitory neurons appear more susceptible to a harsh environment, the ability to preserve their function after intracortical microelectrode implantation may be important to chronic recording outcomes.

After sorting the units for neural recording analysis using MATLAB, the time interval between the trough and peak of the waveform of each single unit was measured to define the repolarization rate^85,143^. The calculated time intervals between peak depolarization and repolarization followed a bimodal distribution (Fig. 2a). Units with a time interval ≤0.40 ms were classified as fast-repolarizing or putative inhibitory neurons, and those with a time interval of >0.40 ms were classified as regular-repolarizing or putative excitatory neurons. Units were then categorized into regular-repolarizing and fast-repolarizing groups across acute (weeks 1–5), subchronic (weeks 6–11), and chronic (week 12) time points for each probe type. The total number of fast-repolarizing and regular-repolarizing units were normalized to the total number of electrode sites, similar to the active electrode yield calculation. The data were normally distributed as determined through a Shapiro–Wilk test. A two-way ANOVA was used to test for significance between probe types within each implant phase. Statistical significance is denoted by * for *p* < 0.05, ** for *p* < 0.01, and *** for *p* < 0.001.

### Differential gene expression analysis

Differential gene expression analysis was used to determine the levels of gene expression related to oxidative stress and neuroinflammation, as described in previous work in the lab^78,82^. At the designated time point of 4 or 12 weeks, rats were perfused with 800–1000 mL of 1X PBS, followed by 500 mL of 30% sucrose in 1X PBS. The brains were extracted and equilibrated in the 30% sucrose in 1X PBS solution for 24 h. Brains were then embedded in OCT, flash-frozen on dry ice, and stored in a −80 °C freezer until cryosectioning. Tissue was sliced into eight sections with a thickness of 150 μm, and a 1 mm diameter biopsy punch centered around the implant site was collected. The tissue from the biopsy punches was placed in a sterile homogenizing tube (19–627, Soft Tissue Homogenizing Mix 1.4 mm Ceramic 2 mL Tubes, Omni International, Kennesaw GA, United States). RNA from the tissue was isolated at the Case Western Reserve University Translational Shared Resource Core Facility using the RNeasy Plus Universal Mini Kit (73404, Qiagen, Hilden, Germany). The nCounter MAX/FLEX system (NanoString Technologies, Seattle, WA, United States) was used to analyze the isolated RNA for counts for a custom codeset of 146 genes, shown in Table 1. Positive and negative control probes were spiked into the isolated RNA from each sample, which was then hybridized with capture and reporter code sets and incubated for 18 h at 65 °C in a thermal cycler. The samples were then analyzed in nCounter Digital Analyzer by scanning the fluorescent reporter probes associated with a particular gene, which then output the counts for each of the 146 genes in the panel.

**Table 1:** Genes analyzed in the in vivo study

Neuronal function	Oxidative stress				Neuroinflammation		Housekeeping
*Bdnf* *Cim* *Cln8* *Dnm2* *Ehd2* *Gnao1* *Gpr37* *Mapt* *Nr4a2* *Psen1* *Snca* *Spp1* *Stx2* *Tor1a* *Tpm1*	*Abl1* *Ager* *Apoe* *App* *Atf4* *Atp13a2* *Atp7a* *Atrn* *Bad* *Bnip3* *Ccl5* *Ccs* *Cdk2* *Cybb* *Cycs* *Ddit3* *Ep300* *Ercc6* *Fas* *Fn1*	*Fxn* *Gsk3b* *Gsr* *Gss* *Gsta1* *Gstm2* *Gstp1* *Gucy1b3* *H2-t23* *Hdac2* *Hdac6* *Hif1a* *Hmox1* *Hspb1* *Htra2* *Idh1* *Ins2* *Ipcef1* *Keap1*	*Lpo* *Lrrk2* *Mgmt* *Mmp14* *Mutyh* *Ncf1* *Nefh* *Nfe2l2* *Nme5* *Nol3* *Nos1* *Nos3* *Noxa1* *Nqo1* *Nr2f6* *Osgin1* *Oxr1* *Park7* *Pink1*	*Pla2g4a* *Ppargc1a* *Prnp* *Scd1* *Serpina3n* *Sirt1* *Sirt2* *Slc8a1* *Sod1* *Sod2* *Sod3* *Src* *Srxn1* *Trp53* *Trpm2* *Txnl1* *Txnrd1* *Ubqln1* *Xbp1*	*Aif1* *Aim2* *Akt1* *Arc* *Bcl2* *Blnk* *C3* *C3ar1* *C4a* *C5ar1* *Casp3* *Casp8* *Cd14* *Cd36* *Cd45* *Cd68* *Cd74* *Cd84* *Clec7a* *Ctss* *Dock2* *Fcer1g* *Fcgr2b* *Fos* *Gfap* *Hgf*	*Il1b* *Il1r1* *Il2rg* *Il6* *Irak4* *Irf7* *Itgam* *Jun* *Lilrb4a* *Mmp12* *Mpeg1* *Ngfr* *Osmr* *Parp1* *Pdgfrb* *Psmb8* *Ptgs2* *Ptpn6* *Ptx3* *Rela* *Tnf* *Tnfrsf1a* *Tnfrsf25* *Tyrobp* *Vegfa*	*Hprt* *Rp113a* *Rps18* *Sdha* *Tbp* *Ubc*

The panel consisted of genes related to neuronal function, oxidative stress, neuroinflammation, as well as housekeeping genes used for normalization

The raw count files were uploaded into the nSolver 4.0 software (Bruker Spatial Biology, Seattle, WA, United States) to analyze the gene expression data. Normalization to housekeeping genes and positive control probes was completed to account for variation from RNA concentration and probe hybridization. Normalized counts were then run through Advanced Analysis 2.0 to determine differential expression using a simplified negative binomial model. A Benjamini–Hochberg correction factor with a false discovery rate of 0.2 was used to reduce the occurrence of false positives. Data was prepared for visualization using GraphPad Prism.

### Immunohistochemistry

Flash frozen cryopreserved tissue slices (20 µm-thick) taken from between the 150 µm tissue slices used for bulk gene expression analysis and placed on glass slides for immunohistochemistry analysis. To prepare for staining, slides were allowed to return to room temperature in a humidity-controlled environment to prevent dehydration. The tissue was then post-fixed with 10% formalin for 15 min followed by three washes with 1X PBS and permeabilization in 1X PBS with 0.1% Triton-X 100 (Sigma) (1X PBS-T) for 15 min. Goat serum blocking buffer (4% v/v serum (Invitrogen), 0.3% v/v Triton-X 100, 0.1% w/v sodium azide (Sigma)) was used to block nonspecific binding of tissue. The tissue was then incubated in primary antibody solution (rabbit anti-glial fibrillary acidic protein (GFAP) 1:500 (Invitrogen) and mouse anti-neuronal nuclei (NeuN) 1:250 (Invitrogen) diluted in goat blocking buffer) overnight at 4 °C. On the following day, the samples were washed 5 times for 3 min each with 1×PBS-T, then incubated for 2 h at room temperature with secondary antibody solution (goat anti-rabbit Alexa Fluor 640 (Abcam) (1:1000) and goat anti-mouse Alexa Fluor 488 (Invitrogen) (1:1000)). The tissue was washed again 5 times for 3 min each with 1×PBS-T. To reduce tissue autofluorescence, 0.5 mM copper sulfate (in DI water) (Sigma) was added for 10 min. Tissue was rinsed with DI water and mounted with DAPI Fluoromount-G (Southern Biotech) on microscope slides for imaging.

Slides were imaged using a 20X objective on a Nikon AR1+ confocal microscope. Nikon Imaging Software- Elements was used to capture and stitch 9 images around the implant site so as to provide a wider field of view without compromising resolution. Exposure times were held consistent and the laser settings were as follows: DAPI laser intensity 2.4, gain 40; GFP laser intensity 3.0, gain 35; Cy5 laser intensity 2.5, gain 30; with a pinhole of 1.2 and offset of 40. Images were averaged 16 times and denoised using Nikon’s Denoise.ai. The implant site was identified via gaps in DAPI signal, indicating lack of cell presence, and verified by astrocyte (GFAP) accumulation about the edge of the hole. Representative images have been slightly enhanced for viewing purposes consistent across all images.

### Correlation of active electrode yield and gene expression

The relationship between gene expression and neural recording functionality was examined by Pearson correlation of the log_2_(fold change) over the mean naïve control expression levels and active electrode yield during the final week for each implant. For this analysis, log_2_(fold change) of individual data points was determined for each gene from the ratio of the normalized counts for each sample and the geometric mean for the naïve control samples. For NeuroNexus probes, only the deepest 8 microelectrode contacts were included in the analysis. The Python package SciPy was used to determine the Pearson correlation, *p* value from a two-sided *t*-test, and the linear regression slope. Statistical significance is denoted by * for *p* < 0.05, ** for *p* < 0.01, and *** for *p* < 0.001. No correction for multiple comparisons was made, which would have yielded no significantly correlated genes. Important GO terms were identified using the Database for Annotation, Visualization and Integrated Discovery (DAVID) Functional Annotation Chart^103,104^.

## Supplementary information

Supplementary Material Revised.

## Data Availability

The datasets used and/or analyzed during the current study will be made available from the corresponding authors upon reasonable request.

## Code availability

The MATLAB code used to analyze the neural recording data will be made available from the corresponding authors upon reasonable request.
