# Supplementary material for: Mechanically-adaptive, resveratrol-eluting neural probes for improved intracortical recording performance and stability
Source: Npj Flex Electron. 2025 Jul 9;9(1):64. doi: 10.1038/s41528-025-00440-5 (PMC12240817; doi:10.1038/s41528-025-00440-5)
Supplement: Supplementary file 1 — Supplementary Material Revised. [file 41528_2025_440_MOESM1_ESM.pdf]

Mechanically-Adaptive, Resveratrol-Eluting Neural Probes for Improved Intracortical Recording Performance and Stability

Natalie N Mueller[1,2], Mali Ya Mungu Ocoko[1,2], Youjoung Kim[1,2], Kate Li[1,2], Kaela Gisser[1,2], Gabriele Glusauskas[1,2], Isabella Lugo[1,2], Peter Dernelle[1,2], Anna Clarissa Hermoso[1,2], Jaime Wang[1,2], Jonathan Duncan[1,2], Lindsey N. Druschel[1,2], Francine Graham[1,2], Jeffrey R. Capadona[1,2], Allison Hess-Dunning[1,2]

[1]Advanced Platform Technology Center, VA Northeast Ohio Healthcare System, Cleveland, Ohio, 44106, United States
[2]Department of Biomedical Engineering, Case Western Reserve University, Cleveland, Ohio, 44106, United States

Supplementary Information

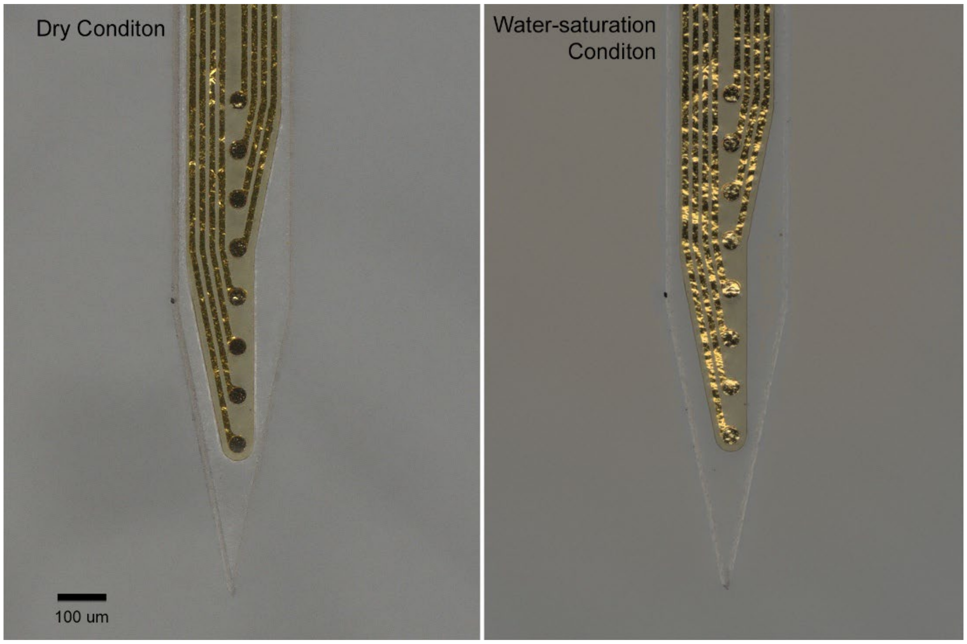

Figure S1. MARE probe in dry condition after microfabrication processing (left); in water-saturated condition while submerged under 2 mm of phosphate buffered saline (PBS) for 20 minutes (right). Little change is noted under these conditions. The swelling from water-saturation slightly dulls the probe tip. The metal and parylene layers remain intact. The metal traces appear to be less flat, which can be attributed to swelling of the polymer nanocomposite substrate (NC) and due to new reflections caused by the surface of the PBS. 0.0001.

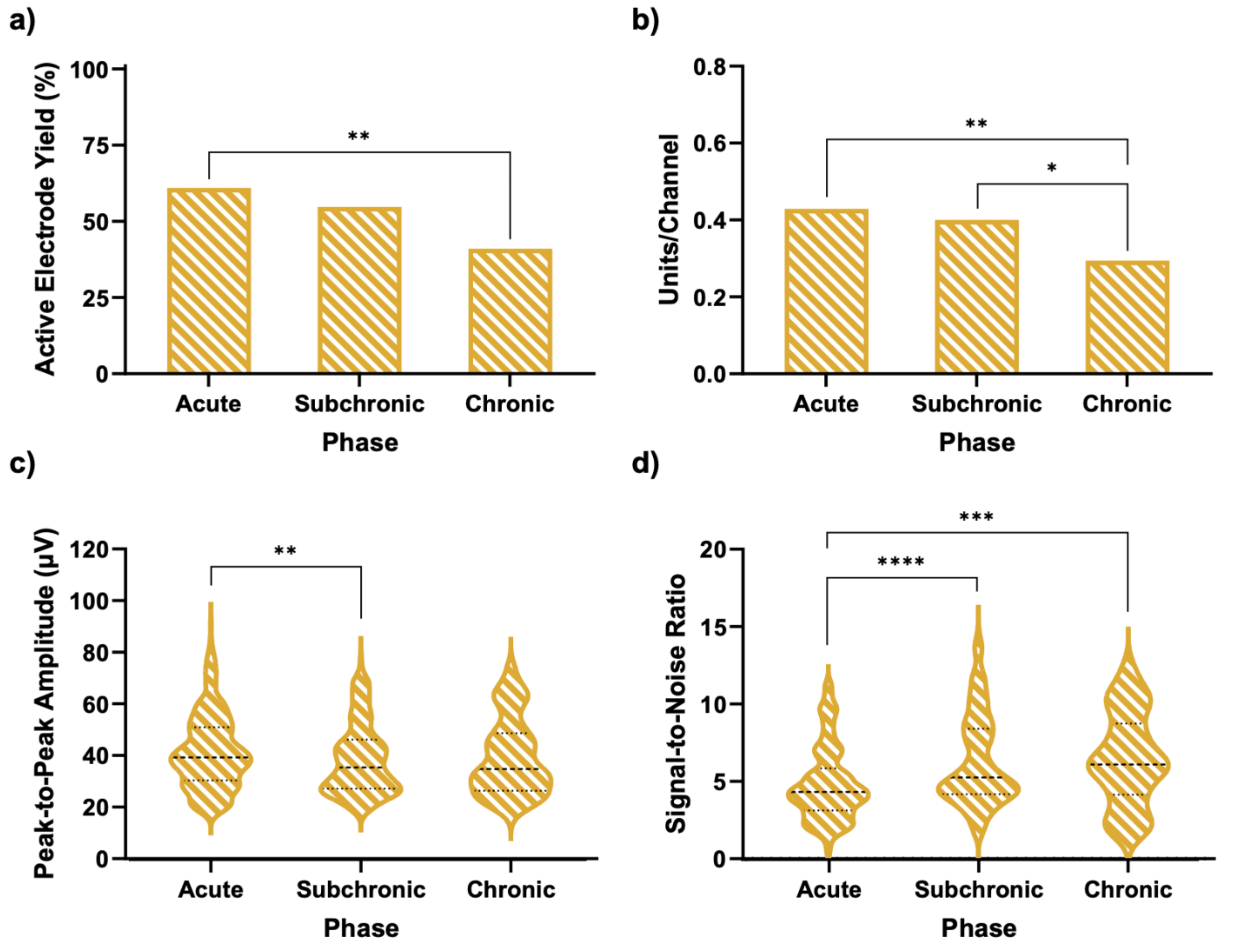

Figure S2. Recording metrics for MA material control probes at the acute, subchronic, and chronic time points. a) shows the active electrode yield, b) shows the units per channel, c) shows the peak-to-peak amplitude, and d) shows the signal-to-noise ratio. Error bars represent standard error. Significance is denoted by * for $p < 0.05$, ** for $p < 0.01$, *** for $p < 0.001$, and **** for $p < 0.0001$.

Table S1. P-values of active electrode yield comparisons across NeuroNexus, MARE, and MA probe types and acute, subchronic, and chronic phases. Significant p-values are shown in colored text, with the color corresponding to the higher active electrode yield in that comparison. P-values in black text are not significant.

		**NeuroNexus**			**MARE**			**MA**		
		**Acute**	**Subchronic**	**Chronic**	**Acute**	**Subchronic**	**Chronic**	**Acute**	**Subchronic**	**Chronic**
**NeuroNexus**	**Acute**		0.0010	0.0004	0.8699			0.0705		
	**Subchronic**			0.0912		0.0023			0.9815	
	**Chronic**						0.3887			1.000
**MARE**	**Acute**					0.7705	0.0216	0.0360		
	**Subchronic**						0.0403		0.0016	
	**Chronic**									0.3435
**MA**	**Acute**								0.1097	0.0075
	**Subchronic**									0.0792
	**Chronic**									

Table S2. P-values of active unit per channel comparisons across NeuroNexus, MARE, and MA probe types and acute, subchronic, and chronic phases. Significant p-values ($p < 0.05$) are shown in colored text, with the color corresponding to the higher units per channel calculation in that comparison. P-values in black text are not significant.

		**NeuroNexus**			**MARE**			**MA**		
		**Acute**	**Subchronic**	**Chronic**	**Acute**	**Subchronic**	**Chronic**	**Acute**	**Subchronic**	**Chronic**
**NeuroNexus**	**Acute**		<0.0001	<0.0001	0.1777			0.0007		
	**Subchronic**			0.0565		0.0001			0.8672	
	**Chronic**						0.5657			1.0000
**MARE**	**Acute**					0.4548	0.0069	0.0411		
	**Subchronic**						0.0023		0.0003	
	**Chronic**									0.5657
**MA**	**Acute**								0.2882	0.0092
	**Subchronic**									0.0431
	**Chronic**									

Table S3. P-values of peak-to-peak amplitude comparisons across NeuroNexus, MARE, and MA probe types and acute, subchronic, and chronic phases. Significant p-values are shown in colored text, with the color corresponding to the higher average peak-to-peak amplitude in that comparison. P-values in black text are not significant.

		NeuroNexus			MARE			MA		
		Acute	Subchronic	Chronic	Acute	Subchronic	Chronic	Acute	Subchronic	Chronic
NeuroNexus	Acute		0.0002	0.0042	0.2966			0.5946		
	Subchronic			0.2338		0.0586			0.5939	
	Chronic						0.0372			0.2077
MARE	Acute					0.0029	0.5538	0.1211		
	Subchronic						0.4868		0.1771	
	Chronic									0.4313
MA	Acute								0.008	0.3392
	Subchronic									0.8182
	Chronic									

Table S4. P-values of signal-to-noise ratio comparisons across NeuroNexus, MARE, and MA probe types and acute, subchronic, and chronic phases. Significant p-values are shown in colored text, with the color corresponding to the higher average signal-to-noise ratio in that comparison. P-values in black text are not significant.

		NeuroNexus			MARE			MA		
		Acute	Subchronic	Chronic	Acute	Subchronic	Chronic	Acute	Subchronic	Chronic
NeuroNexus	Acute		0.5054	0.4766	<0.0001			<0.0001		
	Subchronic			0.6776		0.0977			0.3095	
	Chronic						0.7287			0.8679
MARE	Acute					<0.0001	0.0004	0.7584		
	Subchronic						0.4357		0.5413	
	Chronic									0.8604
MA	Acute								<0.0001	0.0003
	Subchronic									0.4983
	Chronic									

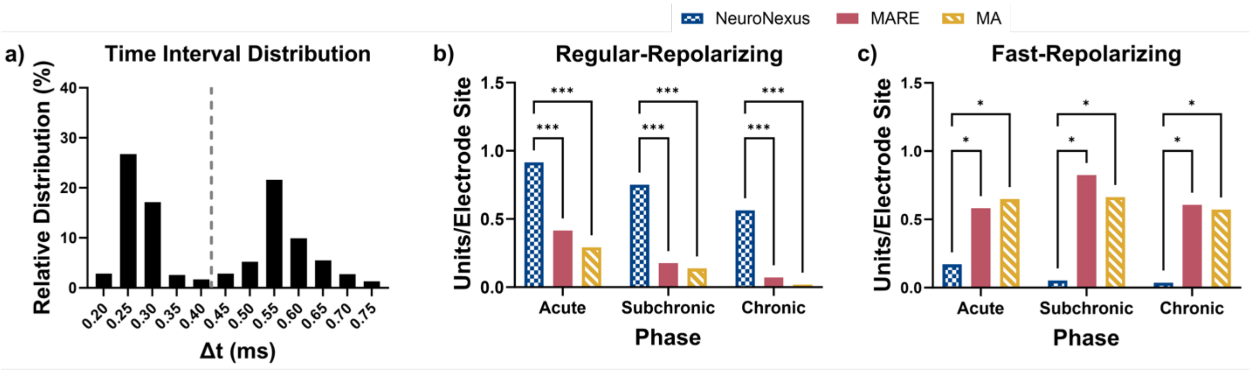

Figure S3. Repolarization rate analysis. a) Histogram showing the relative distribution of the time interval between depolarization and repolarization for all units detected across NeuroNexus, MARE, and MA probes for the 12-week study. The dashed line indicates the threshold between fast-repolarizing and regular-repolarizing. b) The number of regular-repolarizing units/electrode site was significantly higher for NeuroNexus probes compared to MARE and MA probes for the acute, subchronic, and chronic phases. c) The number of fast-repolarizing units/electrode site was significantly higher for MARE and MA probes compared to NeuroNexus probes across all phases. Bars represent the ratio of units recorded divided by the total number of electrode sites. Significance is denoted by * for $p < 0.05$ and *** for $p < 0.001$.

Table S5. Geometric means and geometric standard deviation factors of the impedance magnitude (|Z|) at 1 kHz by week for NeuroNexus, MARE, and MA probes.

		Probe Type					
		NeuroNexus		MARE		MA	
		Geometric mean (kΩ)	Geometric SD factor	Geometric mean (kΩ)	Geometric SD factor	Geometric mean (kΩ)	Geometric SD factor
Week	1	1029.9	1.9	581.0	2.6	489.3	2.9
	2	830.5	2.2	618.6	2.5	568.8	3.0
	3	813.6	2.0	727.7	2.7	534.8	2.7
	4	764.8	1.9	635.1	2.5	449.9	2.6
	5	829.3	1.9	650.3	2.7	505.1	2.7
	6	814.2	1.9	580.2	2.6	457.9	2.6
	7	724.6	1.7	558.3	2.6	413.5	2.7
	8	622.8	1.9	499.9	2.6	412.1	2.5
	9	621.9	1.9	454.9	2.7	364.2	2.5
	10	571.4	1.8	448.5	2.6	348.7	2.4
	11	519.2	1.8	409.3	2.5	309.8	2.2
	12	473.4	2.2	397.6	2.6	337.8	2.6

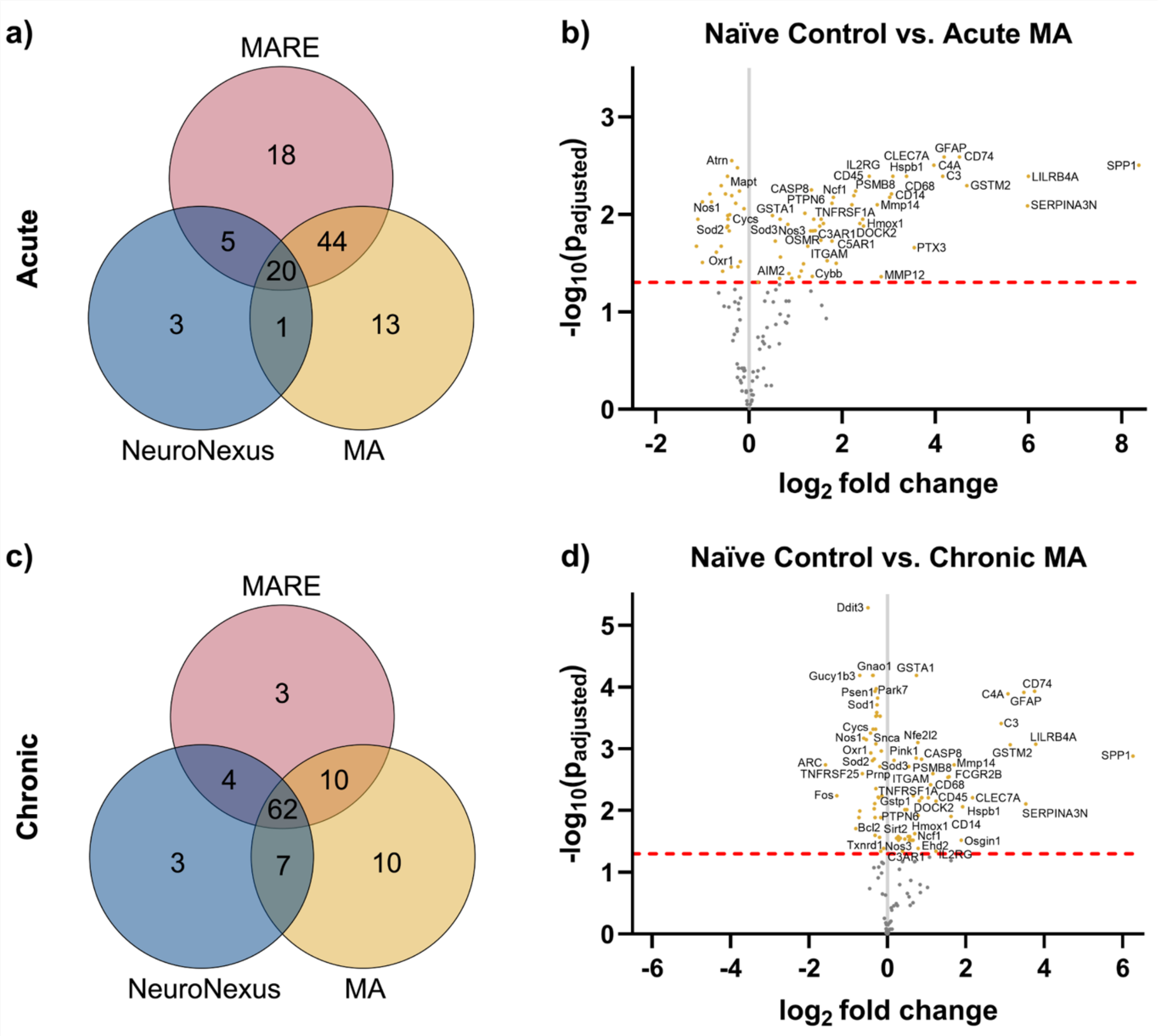

Figure S4. Differential expression for the MA probes vs naïve control at an acute time point of 4 weeks and a chronic time point of 12 weeks. a) Venn diagram showing the number of significant differentially expressed genes between the groups in the acute phase, not accounting for upregulation vs downregulation. b) Volcano plot showing the naïve control vs. the acute MA group, where each point is a gene in the panel. c) Venn diagram showing the number of significant differentially expressed genes between the groups in the chronic phase, not accounting for upregulation vs downregulation. b) Volcano plot showing the naïve control vs. the chronic MA group, where each point is a gene in the panel. The dashed red line shows the significance threshold of $p_{adjusted} < 0.05$. Significant differentially expressed genes are labeled and shown in color. Some significant genes were not labeled due to space limitations. Genes with a $\log_2$ fold change > 0 are upregulated from naïve control, and genes with a $\log_2$ fold change < 0 are downregulated from naïve control.

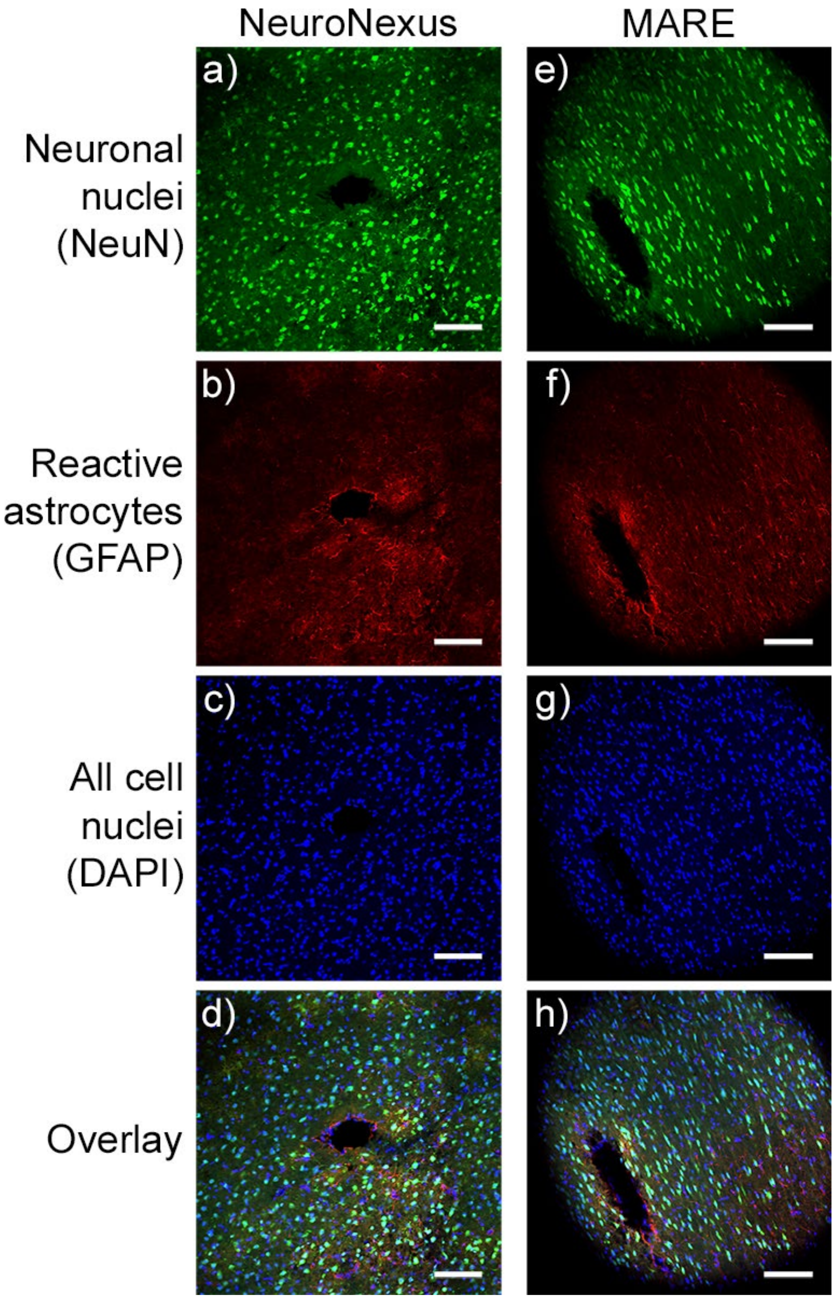

Figure S5. Representative cross-sections of a NeuroNexus (a-d) and a MARE (e-h) implant site from the 12-week implant groups, stained using immunohistochemistry for neuronal nuclei (NeuN, a and e), activated astrocytes (GFAP, b and f); and all cell nuclei (DAPI, c and g). Merged overlays are shown in (d) and (h). The implant locations are represented by the holes left in the tissue. Notably, a high density of neurons is visible near the device-tissue interface (at the edge of the hole) for the MARE sample, while there is a notable lack of neurons in the tissue closest to the implant site for the NeuroNexus sample. The intensity of the GFAP expression appears to be high around the edge of the NeuroNexus implant site, and more diffuse around the MARE probe. While other cell types, likely activated microglia and macrophages, are present near the interface, a dense glial scar is absent around the MARE probe. The immunohistochemistry observations are consistent with gene expression results indicating reduced neuroinflammation at the chronic time point in the MARE group, and they support the sustained recording performance observed in the subchronic and chronic phases for the MARE probes. The scale bar is 100 µm.

Table S6. List of analyzed genes describing if the gene was upregulated, downregulated, or not significant in all comparisons shown in volcano plots based on figure number.

Gene	Fig. 4b	Fig. 4c	Fig. 5b	Fig. 5c	Fig. 6a	Fig. 6b	Fig. S3b	Fig. S3d
Abl1	Not Significant	Not Significant	Not Significant	Not Significant	Not Significant	Not Significant	Not Significant	Not Significant
Ager	Not Significant	Not Significant	Not Significant	Not Significant	Not Significant	Not Significant	Not Significant	Not Significant
Aif1	Not Significant	Upregulated	Not Significant	Not Significant	Not Significant	Not Significant	Upregulated	Upregulated
Aim2	Not Significant	Upregulated	Upregulated	Not Significant	Not Significant	Not Significant	Upregulated	Not Significant
Akt1	Not Significant	Not Significant	Downregulated	Downregulated	Not Significant	Not Significant	Not Significant	Not Significant
Apoe	Not Significant	Upregulated	Upregulated	Upregulated	Not Significant	Not Significant	Not Significant	Upregulated
App	Not Significant	Downregulated	Downregulated	Downregulated	Not Significant	Not Significant	Downregulated	Downregulated
Arc	Not Significant	Downregulated	Downregulated	Downregulated	Not Significant	Downregulated	Downregulated	Downregulated
Atf4	Downregulated	Downregulated	Downregulated	Downregulated	Not Significant	Not Significant	Not Significant	Downregulated
Atp13a2	Downregulated	Downregulated	Downregulated	Downregulated	Not Significant	Not Significant	Downregulated	Downregulated
Atp7a	Not Significant	Not Significant	Not Significant	Not Significant	Not Significant	Not Significant	Not Significant	Not Significant
Atrn	Downregulated	Downregulated	Downregulated	Downregulated	Not Significant	Not Significant	Downregulated	Downregulated
Bad	Not Significant	Not Significant	Downregulated	Not Significant	Not Significant	Not Significant	Not Significant	Downregulated
Bcl2	Not Significant	Not Significant	Downregulated	Downregulated	Not Significant	Downregulated	Not Significant	Downregulated
Bdnf	Not Significant	Not Significant	Downregulated	Downregulated	Not Significant	Not Significant	Downregulated	Downregulated
Blnk	Not Significant	Not Significant	Not Significant	Not Significant	Not Significant	Not Significant	Not Significant	Upregulated
Bnip3	Not Significant	Not Significant	Not Significant	Not Significant	Not Significant	Not Significant	Not Significant	Not Significant
C3	Not Significant	Upregulated	Upregulated	Upregulated	Not Significant	Not Significant	Upregulated	Upregulated
C3ar1	Not Significant	Upregulated	Not Significant	Upregulated	Not Significant	Not Significant	Upregulated	Upregulated
C4a	Not Significant	Upregulated	Upregulated	Upregulated	Not Significant	Not Significant	Upregulated	Upregulated
C5ar1	Not Significant	Not Significant	Not Significant	Not Significant	Not Significant	Not Significant	Upregulated	Not Significant
Casp3	Not Significant	Upregulated	Not Significant	Not Significant	Not Significant	Not Significant	Not Significant	Not Significant
Casp8	Not Significant	Upregulated	Upregulated	Upregulated	Not Significant	Not Significant	Upregulated	Upregulated
Ccl5	Not Significant	Not Significant	Not Significant	Not Significant	Not Significant	Not Significant	Not Significant	Not Significant
Ccs	Not Significant	Upregulated	Not Significant	Not Significant	Not Significant	Downregulated	Not Significant	Not Significant
Cd14	Not Significant	Upregulated	Not Significant	Upregulated	Not Significant	Not Significant	Upregulated	Upregulated
Cd36	Not Significant	Upregulated	Not Significant	Not Significant	Not Significant	Downregulated	Upregulated	Not Significant
Cd45	Not Significant	Upregulated	Upregulated	Upregulated	Not Significant	Not Significant	Upregulated	Upregulated
Cd68	Not Significant	Upregulated	Upregulated	Upregulated	Not Significant	Not Significant	Upregulated	Upregulated
Cd74	Not Significant	Upregulated	Upregulated	Upregulated	Not Significant	Not Significant	Upregulated	Upregulated
Cd84	Not Significant	Not Significant	Not Significant	Not Significant	Not Significant	Not Significant	Upregulated	Not Significant
Cdk2	Not Significant	Upregulated	Not Significant	Upregulated	Not Significant	Not Significant	Upregulated	Not Significant
Cim	Not Significant	Upregulated	Not Significant	Not Significant	Not Significant	Not Significant	Upregulated	Upregulated
Clec7a	Not Significant	Upregulated	Upregulated	Upregulated	Not Significant	Not Significant	Upregulated	Upregulated
Cln8	Not Significant	Not Significant	Not Significant	Not Significant	Not Significant	Not Significant	Not Significant	Not Significant
Ctss	Not Significant	Not Significant	Upregulated	Not Significant	Not Significant	Not Significant	Not Significant	Upregulated
Cybb	Not Significant	Not Significant	Not Significant	Not Significant	Not Significant	Not Significant	Upregulated	Not Significant
Cycs	Not Significant	Downregulated	Downregulated	Downregulated	Not Significant	Not Significant	Downregulated	Downregulated
Ddit3	Not Significant	Downregulated	Downregulated	Downregulated	Not Significant	Downregulated	Not Significant	Downregulated
Dnm2	Upregulated	Upregulated	Upregulated	Upregulated	Not Significant	Not Significant	Upregulated	Upregulated
Dock2	Not Significant	Upregulated	Upregulated	Upregulated	Not Significant	Not Significant	Upregulated	Upregulated
Ehd2	Upregulated	Upregulated	Not Significant	Not Significant	Not Significant	Downregulated	Upregulated	Upregulated
Ep300	Downregulated	Downregulated	Downregulated	Downregulated	Not Significant	Not Significant	Downregulated	Downregulated
Ercc6	Not Significant	Not Significant	Not Significant	Not Significant	Not Significant	Not Significant	Downregulated	Not Significant
Fas	Not Significant	Not Significant	Not Significant	Not Significant	Not Significant	Not Significant	Not Significant	Not Significant
Fcer1g	Not Significant	Upregulated	Not Significant	Not Significant	Not Significant	Not Significant	Upregulated	Upregulated
Fcgr2b	Not Significant	Upregulated	Upregulated	Upregulated	Not Significant	Not Significant	Upregulated	Upregulated
Fn1	Not Significant	Upregulated	Not Significant	Not Significant	Not Significant	Not Significant	Upregulated	Upregulated
Fos	Not Significant	Not Significant	Not Significant	Downregulated	Not Significant	Downregulated	Not Significant	Downregulated
Fxn	Not Significant	Not Significant	Downregulated	Downregulated	Not Significant	Not Significant	Not Significant	Not Significant
Gfap	Upregulated	Upregulated	Upregulated	Upregulated	Not Significant	Downregulated	Upregulated	Upregulated
Gnao1	Downregulated	Downregulated	Downregulated	Downregulated	Not Significant	Not Significant	Downregulated	Downregulated
Gpr37	Not Significant	Upregulated	Upregulated	Upregulated	Not Significant	Not Significant	Not Significant	Upregulated
Gsk3b	Downregulated	Downregulated	Downregulated	Downregulated	Not Significant	Upregulated	Downregulated	Downregulated
Gsr	Not Significant	Not Significant	Not Significant	Not Significant	Not Significant	Not Significant	Not Significant	Not Significant
Gss	Not Significant	Not Significant	Not Significant	Not Significant	Not Significant	Not Significant	Not Significant	Not Significant
Gsta1	Upregulated	Upregulated	Upregulated	Upregulated	Not Significant	Not Significant	Upregulated	Upregulated
Gstm2	Upregulated	Upregulated	Upregulated	Upregulated	Not Significant	Not Significant	Upregulated	Upregulated
Gstp1	Not Significant	Not Significant	Upregulated	Upregulated	Not Significant	Not Significant	Not Significant	Upregulated
Gucy1b3	Downregulated	Downregulated	Downregulated	Downregulated	Not Significant	Not Significant	Downregulated	Downregulated
H2-t23	Not Significant	Not Significant	Not Significant	Not Significant	Not Significant	Not Significant	Not Significant	Not Significant
Hdac2	Not Significant	Not Significant	Not Significant	Not Significant	Not Significant	Not Significant	Downregulated	Downregulated
Hdac6	Not Significant	Not Significant	Downregulated	Downregulated	Not Significant	Not Significant	Not Significant	Downregulated
Hgf	Downregulated	Not Significant	Downregulated	Downregulated	Not Significant	Not Significant	Downregulated	Downregulated
Hif1a	Not Significant	Not Significant	Not Significant	Not Significant	Not Significant	Not Significant	Downregulated	Not Significant
Hmox1	Not Significant	Upregulated	Not Significant	Upregulated	Not Significant	Not Significant	Upregulated	Upregulated
Hspb1	Upregulated	Upregulated	Not Significant	Upregulated	Not Significant	Downregulated	Upregulated	Upregulated
Htra2	Not Significant	Downregulated	Downregulated	Downregulated	Not Significant	Not Significant	Not Significant	Downregulated
Idh1	Not Significant	Not Significant	Not Significant	Not Significant	Not Significant	Not Significant	Not Significant	Not Significant

Gene	Fig. 4b	Fig. 4c	Fig. 5b	Fig. 5c	Fig. 6a	Fig. 6b	Fig. S3b	Fig. S3d
Il1b	Not Significant	Upregulated	Not Significant	Upregulated	Not Significant	Not Significant	Upregulated	Not Significant
Il1r1	Not Significant	Upregulated	Not Significant	Upregulated	Not Significant	Downregulated	Upregulated	Upregulated
Il2rg	Not Significant	Upregulated	Not Significant	Upregulated	Not Significant	Not Significant	Upregulated	Upregulated
Il6	Not Significant	Not Significant	Not Significant	Not Significant	Not Significant	Not Significant	Not Significant	
Ins2	Not Significant	Upregulated	Not Significant	Not Significant	Not Significant	Not Significant	Not Significant	Not Significant
Ipcef1	Not Significant	Downregulated	Not Significant	Not Significant	Not Significant	Not Significant	Downregulated	Not Significant
Irak4	Not Significant	Not Significant	Not Significant	Not Significant	Not Significant	Not Significant	Upregulated	Not Significant
Irf7	Not Significant	Upregulated	Not Significant	Not Significant	Not Significant	Not Significant	Not Significant	Not Significant
Itgam	Not Significant	Upregulated	Not Significant	Upregulated	Not Significant	Not Significant	Upregulated	Upregulated
Jun	Not Significant	Not Significant	Not Significant	Downregulated	Not Significant	Downregulated	Not Significant	Not Significant
Keap1	Not Significant	Not Significant	Downregulated	Downregulated	Not Significant	Not Significant	Not Significant	Downregulated
Lilrb4a	Not Significant	Upregulated	Upregulated	Upregulated	Not Significant	Not Significant	Upregulated	Upregulated
Lpo	Not Significant	Not Significant	Not Significant	Not Significant	Not Significant	Not Significant	Not Significant	Not Significant
Lrrk2	Not Significant	Downregulated	Downregulated	Downregulated	Not Significant	Not Significant	Downregulated	Downregulated
Mapt	Downregulated	Downregulated	Downregulated	Downregulated	Not Significant	Not Significant	Downregulated	Downregulated
Mgmt	Not Significant	Upregulated	Not Significant	Not Significant	Not Significant	Not Significant	Not Significant	Not Significant
Mmp12	Not Significant	Upregulated	Not Significant	Not Significant	Not Significant	Not Significant	Upregulated	Not Significant
Mmp14	Upregulated	Upregulated	Upregulated	Upregulated	Not Significant	Downregulated	Upregulated	Upregulated
Mpeg1	Not Significant	Not Significant	Not Significant	Not Significant	Not Significant	Not Significant	Not Significant	Not Significant
Mutyh	Not Significant	Not Significant	Not Significant	Not Significant	Not Significant	Not Significant	Not Significant	Not Significant
Ncf1	Not Significant	Upregulated	Not Significant	Not Significant	Not Significant	Not Significant	Upregulated	Upregulated
Nefh	Not Significant	Downregulated	Not Significant	Not Significant	Not Significant	Not Significant	Not Significant	Not Significant
Nfe2l2	Upregulated	Upregulated	Upregulated	Upregulated	Not Significant	Not Significant	Not Significant	Upregulated
Ngfr	Not Significant	Upregulated	Upregulated	Upregulated	Not Significant	Not Significant	Not Significant	Not Significant
Nme5	Not Significant	Not Significant	Downregulated	Downregulated	Not Significant	Not Significant	Not Significant	Downregulated
Nol3	Not Significant	Not Significant	Not Significant	Not Significant	Not Significant	Not Significant	Not Significant	Not Significant
Nos1	Downregulated	Downregulated	Downregulated	Downregulated	Not Significant	Not Significant	Downregulated	Downregulated
Nos3	Not Significant	Upregulated	Not Significant	Not Significant	Not Significant	Not Significant	Upregulated	Upregulated
Noxa1	Not Significant	Not Significant	Not Significant	Not Significant	Not Significant	Not Significant	Not Significant	Not Significant
Nqo1	Not Significant	Upregulated	Not Significant	Not Significant	Not Significant	Not Significant	Not Significant	Upregulated
Nr2f6	Not Significant	Not Significant	Not Significant	Not Significant	Not Significant	Not Significant	Not Significant	Not Significant
Nr4a2	Not Significant	Not Significant	Not Significant	Not Significant	Not Significant	Not Significant	Not Significant	Not Significant
Osgin1	Not Significant	Upregulated	Upregulated	Upregulated	Not Significant	Not Significant	Upregulated	Upregulated
Osmr	Not Significant	Upregulated	Not Significant	Not Significant	Not Significant	Downregulated	Upregulated	Not Significant
Oxr1	Downregulated	Downregulated	Downregulated	Downregulated	Not Significant	Not Significant	Downregulated	Downregulated
Park7	Downregulated	Not Significant	Downregulated	Downregulated	Not Significant	Not Significant	Not Significant	Downregulated
Parp1	Downregulated	Downregulated	Downregulated	Downregulated	Not Significant	Not Significant	Not Significant	Downregulated
Pdgfrb	Not Significant	Upregulated	Not Significant	Not Significant	Not Significant	Downregulated	Not Significant	Not Significant
Pink1	Not Significant	Not Significant	Upregulated	Upregulated	Not Significant	Not Significant	Not Significant	Upregulated
Pla2g4a	Not Significant	Upregulated	Upregulated	Not Significant	Not Significant	Not Significant	Not Significant	Upregulated
Ppargc1a	Not Significant	Downregulated	Downregulated	Downregulated	Not Significant	Not Significant	Not Significant	Downregulated
Prnp	Not Significant	Downregulated	Downregulated	Downregulated	Not Significant	Not Significant	Downregulated	Downregulated
Psen1	Not Significant	Not Significant	Downregulated	Downregulated	Downregulated	Downregulated	Downregulated	Downregulated
Psmb8	Not Significant	Upregulated	Upregulated	Upregulated	Not Significant	Not Significant	Upregulated	Upregulated
Ptgs2	Not Significant	Not Significant	Downregulated	Downregulated	Not Significant	Not Significant	Downregulated	Downregulated
Ptpn6	Not Significant	Upregulated	Upregulated	Not Significant	Not Significant	Not Significant	Upregulated	Upregulated
Ptx3	Not Significant	Upregulated	Upregulated	Not Significant	Not Significant	Downregulated	Upregulated	Not Significant
Rela	Not Significant	Upregulated	Upregulated	Not Significant	Not Significant	Not Significant	Upregulated	Upregulated
Scd1	Not Significant	Not Significant	Not Significant	Not Significant	Not Significant	Not Significant	Not Significant	Not Significant
Serpina3n		Upregulated		Upregulated	Not Significant	Downregulated	Upregulated	Upregulated
Sirt1	Not Significant	Not Significant	Not Significant	Not Significant	Not Significant	Not Significant	Not Significant	Not Significant
Sirt2	Not Significant	Upregulated	Upregulated	Not Significant	Not Significant	Not Significant	Not Significant	Upregulated
Slc8a1	Not Significant	Downregulated	Not Significant	Downregulated	Not Significant	Not Significant	Downregulated	Downregulated
Snca	Downregulated	Downregulated	Downregulated	Downregulated	Not Significant	Not Significant	Not Significant	Downregulated
Sod1	Not Significant	Downregulated	Downregulated	Downregulated	Not Significant	Not Significant	Not Significant	Downregulated
Sod2	Downregulated	Downregulated	Downregulated	Downregulated	Not Significant	Not Significant	Downregulated	Downregulated
Sod3	Upregulated	Upregulated	Upregulated	Upregulated	Not Significant	Downregulated	Upregulated	Upregulated
Spp1	Not Significant	Upregulated	Upregulated	Upregulated	Not Significant	Not Significant	Upregulated	Upregulated
Src	Not Significant	Not Significant	Not Significant	Not Significant	Not Significant	Not Significant	Not Significant	Not Significant
Srxn1	Not Significant	Not Significant	Downregulated	Downregulated	Not Significant	Not Significant	Upregulated	Not Significant
Stx2	Not Significant	Not Significant	Not Significant	Not Significant	Not Significant	Not Significant	Not Significant	Not Significant
Tnf	Not Significant	Not Significant		Not Significant	Not Significant	Not Significant	Upregulated	Not Significant
Tnfrsf1a	Not Significant	Upregulated	Upregulated	Upregulated	Not Significant	Not Significant	Upregulated	Upregulated
Tnfrsf25	Not Significant	Not Significant	Downregulated	Downregulated	Not Significant	Downregulated	Not Significant	Downregulated
Tor1a	Downregulated	Downregulated	Downregulated	Downregulated	Not Significant	Not Significant	Downregulated	Downregulated
Tpm1	Downregulated	Not Significant	Downregulated	Downregulated	Not Significant	Downregulated	Not Significant	Downregulated
Trp53	Not Significant	Not Significant	Not Significant	Not Significant	Not Significant	Not Significant	Not Significant	Not Significant
Trpm2	Not Significant	Not Significant	Downregulated	Not Significant	Not Significant	Not Significant	Not Significant	Not Significant
Txnl1	Downregulated	Downregulated	Downregulated	Downregulated	Not Significant	Not Significant	Downregulated	Downregulated
Txnrd1	Downregulated	Not Significant	Downregulated	Downregulated	Not Significant	Not Significant	Not Significant	Downregulated
Tyrobp	Not Significant	Upregulated	Upregulated	Not Significant	Not Significant	Not Significant	Upregulated	Upregulated
Ubqln1	Downregulated	Downregulated	Downregulated	Downregulated	Not Significant	Not Significant	Not Significant	Downregulated
Vegfa	Not Significant	Not Significant	Not Significant	Not Significant	Not Significant	Not Significant	Downregulated	Not Significant
Xbp1	Not Significant	Upregulated	Not Significant	Not Significant	Not Significant	Downregulated	Not Significant	Not Significant

Table S7. List of analyzed genes describing the correlation with active electrode yield for each probe type and end time point, where *r* represents Pearson correlation, *s* represents the sensitivity to the gene in terms of the change in $\log_2$(fold change) required to increase the active electrode yield by one microelectrode site, given the number and spacing of the implants used; *p* is the uncorrected p-value indicating the significance of correlation as determined by a 2-sided t-test. The set of *r, s,* and *p* values for probe groups and end time points with significant correlation to the final week active electrode yield are indicated with bold red font.

	NeuroNexus						MARE						MA					
	Acute			Chronic			Acute			Chronic			Acute			Chronic		
**Gene**	r	s	p	r	s	p	r	s	p	r	s	p	r	s	p	r	s	p
**Abl1**	**1.00**	**0.035**	**0.028**	0.00	N/A	0.999	0.72	0.048	0.279	0.24	N/A	0.597	**1.00**	**0.344**	**0.043**	-0.46	-0.024	0.300
**Ager**	-0.91	-0.141	0.278	0.16	N/A	0.726	0.67	0.115	0.326	-0.04	N/A	0.938	-0.15	N/A	0.905	-0.20	N/A	0.673
**Aif1**	0.98	0.127	0.128	**-0.77**	**-0.119**	**0.043**	0.06	N/A	0.943	0.33	N/A	0.471	0.71	0.228	0.494	-0.37	N/A	0.410
**Aim2**	0.13	N/A	0.919	-0.62	-0.117	0.134	0.55	0.056	0.834	-0.02	N/A	0.970	-0.03	N/A	0.984	0.06	N/A	0.139
**Akt1**	0.88	0.056	0.318	-0.02	N/A	0.966	-0.86	-0.011	0.136	0.29	N/A	0.532	0.99	0.110	0.090	-0.59	-0.022	0.163
**Apoe**	0.88	0.151	0.313	-0.45	-0.079	0.305	-0.07	N/A	0.928	0.59	0.053	0.163	-0.58	-0.161	0.609	-0.28	N/A	0.540
**App**	-0.95	-0.031	0.199	0.64	0.023	0.121	-0.37	N/A	0.630	-0.28	N/A	0.538	0.63	0.027	0.569	0.64	0.021	0.123
**Arc**	-0.58	-0.106	0.604	0.38	N/A	0.398	-0.36	N/A	0.644	-0.02	N/A	0.958	0.99	0.010	2.884	0.33	N/A	1.225
**Atf4**	-0.64	-0.003	0.556	0.56	0.036	0.188	-0.35	N/A	0.650	0.10	N/A	0.825	0.79	0.134	0.417	0.56	0.050	0.193
**Atp13a2**	0.98	0.011	0.142	0.45	0.041	0.315	0.01	N/A	0.992	**-0.87**	**-0.036**	**0.011**	-0.86	-0.198	0.341	-0.10	N/A	0.829
**Atp7a**	0.35	N/A	0.775	0.02	N/A	0.972	0.36	N/A	0.644	-0.08	N/A	0.864	-0.46	-0.090	0.698	0.56	0.087	0.194
**Atrn**	-0.98	-0.028	0.114	0.37	N/A	0.409	-0.06	N/A	0.939	-0.61	-0.028	0.149	0.73	0.029	0.478	**0.85**	**0.029**	**0.016**
**Bad**	0.97	0.041	0.159	-0.45	-0.028	0.307	0.74	0.043	0.262	0.19	N/A	0.679	**1.00**	**0.082**	**0.022**	-0.29	N/A	0.533
**Bcl2**	-0.65	-0.005	0.553	-0.30	N/A	0.518	-0.73	-0.049	0.270	0.57	0.054	0.184	0.13	N/A	0.918	0.56	0.064	0.189
**Bdnf**	-0.47	-0.079	0.685	0.05	N/A	0.911	-0.36	N/A	0.644	0.44	0.071	0.328	-0.59	-0.078	0.595	0.68	0.174	0.094
**Blnk**	0.89	0.150	0.307	-0.42	-0.063	0.343	-0.06	N/A	0.943	0.22	N/A	0.641	-0.52	-0.101	0.650	-0.08	N/A	0.863
**Bnip3**	0.61	0.025	0.579	0.45	0.047	0.316	-0.84	-0.168	0.162	0.43	0.077	0.332	-0.98	-0.270	0.131	-0.25	N/A	0.591
**C3**	0.93	0.381	0.232	-0.52	-0.257	0.231	0.26	N/A	0.741	0.33	N/A	0.470	0.45	0.264	0.704	-0.29	N/A	0.532
**C3ar1**	0.88	0.140	0.321	-0.61	-0.159	0.148	0.28	N/A	0.721	0.32	N/A	0.477	0.69	0.208	0.514	-0.29	N/A	0.532
**C4a**	1.00	0.273	0.061	-0.32	N/A	0.479	0.28	N/A	0.718	0.46	0.332	0.294	-0.21	N/A	0.867	-0.33	N/A	0.471
**C5ar1**	0.90	0.102	0.291	**-0.79**	**-0.195**	**0.034**	0.36	N/A	0.637	0.45	0.233	0.316	0.37	N/A	0.756	-0.41	-0.159	0.360
**Casp3**	0.95	0.040	0.209	**-0.76**	**-0.034**	**0.047**	-0.47	-0.015	0.530	0.40	N/A	0.375	0.64	0.067	0.561	0.41	0.017	0.367
**Casp8**	0.95	0.167	0.196	-0.51	-0.138	0.237	0.31	N/A	0.694	0.48	0.148	0.281	-0.46	-0.106	0.695	-0.51	-0.095	0.248
**Ccl1**	0.64	0.153	0.556	**0.87**	**0.213**	**0.011**	0.13	N/A	0.868	-0.22	N/A	0.634	-0.63	-0.055	0.562	-0.13	N/A	0.780
**Ccl5**	**1.00**	**0.213**	**0.023**	**-0.78**	**-0.522**	**0.039**	0.48	0.181	0.522	0.42	0.253	0.352	-0.53	-0.391	0.645	0.41	0.274	0.355
**Ccs**	0.74	0.015	0.470	-0.40	-0.031	0.370	0.16	N/A	0.843	0.66	0.047	0.103	0.93	0.151	0.240	-0.56	-0.046	0.192
**Cd14**	0.99	0.273	0.107	-0.61	-0.246	0.147	0.26	N/A	0.742	0.58	0.283	0.175	-0.19	N/A	0.877	-0.35	N/A	0.447
**Cd36**	0.98	0.185	0.129	0.04	N/A	0.930	0.54	0.151	0.455	0.62	0.231	0.137	0.86	0.500	0.341	-0.36	N/A	0.430
**Cd45**	0.97	0.242	0.153	-0.51	-0.176	0.237	0.20	N/A	0.802	0.39	N/A	0.386	0.14	N/A	0.908	-0.30	N/A	0.521
**Cd68**	**1.00**	**0.469**	**0.047**	-0.59	-0.349	0.165	0.53	0.333	0.475	0.33	N/A	0.474	0.24	N/A	0.844	-0.38	N/A	0.395
**Cd74**	0.92	0.557	0.255	-0.39	N/A	0.381	0.48	0.269	0.517	0.31	N/A	0.501	-0.06	N/A	0.960	-0.34	N/A	0.452
**Cd84**	0.98	0.138	0.142	-0.14	N/A	0.766	0.09	N/A	0.912	0.20	N/A	0.664	0.12	N/A	0.921	-0.29	N/A	0.533
**Cdk2**	0.52	0.046	0.655	**-0.85**	**-0.090**	**0.015**	0.07	N/A	0.927	0.16	N/A	0.724	-0.35	N/A	0.773	-0.29	N/A	0.535
**Cim**	0.93	0.129	0.243	-0.44	-0.111	0.329	0.13	N/A	0.866	0.50	0.133	0.256	-0.60	-0.276	0.588	-0.15	N/A	0.745
**Clec7a**	0.98	0.353	0.120	-0.56	-0.238	0.192	0.32	N/A	0.675	0.39	N/A	0.392	0.28	N/A	0.817	-0.14	N/A	0.762
**Cln8**	0.88	0.025	0.319	0.12	N/A	0.797	0.10	N/A	0.905	-0.29	N/A	0.533	0.95	0.080	0.200	0.62	0.044	0.139
**Ct22**	0.94	0.126	0.229	-0.58	-0.080	0.170	0.06	N/A	0.944	0.16	N/A	0.735	0.90	0.136	0.281	0.02	N/A	0.960
**Cybb**	0.97	0.132	0.151	0.08	N/A	0.864	0.25	N/A	0.749	0.29	N/A	0.523	0.47	0.160	0.687	-0.27	N/A	0.560
**Cycs**	-0.95	-0.069	0.197	0.38	N/A	0.404	-0.19	N/A	0.805	0.50	0.031	0.259	0.17	N/A	0.890	0.28	N/A	0.538
**Ddit3**	0.59	0.028	0.599	-0.23	N/A	0.624	0.84	0.025	0.159	-0.55	-0.042	0.198	0.52	0.036	0.651	-0.47	-0.017	0.287
**Dnm2**	-0.69	-0.012	0.515	-0.05	N/A	0.923	0.24	N/A	0.762	0.21	N/A	0.646	0.97	0.320	0.158	**-0.81**	**-0.108**	**0.027**
**Dock2**	**1.00**	**0.174**	**0.015**	-0.71	-0.129	0.076	0.14	N/A	0.863	0.25	N/A	0.584	0.13	N/A	0.915	-0.36	N/A	0.423
**Ehd2**	1.00	0.101	0.060	-0.68	-0.116	0.094	-0.03	N/A	0.966	0.52	0.066	0.231	-0.93	-0.483	0.237	-0.20	N/A	0.662
**Ep300**	-0.88	-0.026	0.319	0.12	N/A	0.796	-0.45	-0.010	0.552	-0.42	-0.017	0.351	0.11	N/A	0.928	**0.88**	**0.031**	**0.009**
**Ercc6**	0.06	N/A	0.959	-0.44	-0.013	0.327	-0.41	-0.025	0.592	-0.14	N/A	0.770	-0.47	-0.026	0.686	-0.03	N/A	0.949

	NeuroNexus						MARE						MA					
	Acute			Chronic			Acute			Chronic			Acute			Chronic		
Gene	r	s	p	r	s	p	r	s	p	r	s	p	r	s	p	r	s	p
Fas	0.75	0.128	0.462	-0.46	-0.172	0.300	0.48	0.199	0.518	-0.18	N/A	0.704	0.61	0.117	0.583	-0.19	N/A	0.675
Fcer1g	0.91	0.168	0.266	-0.55	-0.094	0.199	0.20	N/A	0.804	0.32	N/A	0.477	0.67	0.276	0.529	-0.31	N/A	0.500
Fcer2b	0.78	0.240	0.427	-0.61	-0.158	0.148	0.15	N/A	0.847	0.52	0.307	0.232	0.31	N/A	0.796	-0.30	N/A	0.508
Fn1	0.96	0.211	0.184	-0.16	N/A	0.730	0.31	N/A	0.688	0.56	0.150	0.187	-0.20	N/A	0.874	0.13	N/A	0.786
Fos	-0.69	-0.157	0.517	0.09	N/A	0.845	-0.78	-0.158	0.215	0.28	N/A	0.544	0.87	0.130	0.334	0.53	0.222	0.220
Fxn	-0.67	-0.028	0.532	-0.27	N/A	0.551	-0.39	N/A	0.609	-0.02	N/A	0.962	0.96	0.090	0.190	-0.35	N/A	0.446
Gfap	0.97	0.382	0.163	-0.73	-0.451	0.063	0.20	N/A	0.803	0.50	0.248	0.254	0.11	N/A	0.927	-0.23	N/A	0.617
Gnao1	-0.98	-0.055	0.137	-0.12	N/A	0.798	-0.44	-0.032	0.556	-0.42	-0.012	0.345	-0.67	-0.070	0.531	0.71	0.029	0.072
Gpr37	**-1.00**	**-0.185**	**0.046**	-0.13	N/A	0.783	0.69	0.096	0.306	-0.34	N/A	0.457	-0.25	N/A	0.839	0.21	N/A	0.650
Gsk3b	-0.93	-0.012	0.244	0.40	N/A	0.380	-0.28	N/A	0.716	-0.12	N/A	0.790	-0.71	-0.050	0.497	0.39	N/A	0.384
Gsr	-0.88	-0.012	0.316	0.11	N/A	0.810	0.31	N/A	0.693	-0.42	-0.030	0.349	**-1.00**	**-0.269**	**0.032**	-0.49	-0.031	0.266
Gss	-0.28	-0.017	0.819	0.01	N/A	0.976	0.13	N/A	0.873	0.13	N/A	0.782	0.32	N/A	0.796	-0.06	N/A	0.904
Gsta1	0.85	0.090	0.347	-0.58	-0.058	0.169	-0.70	-0.086	0.297	0.74	0.069	0.060	-0.56	-0.046	0.625	-0.22	N/A	0.633
Gsta2	0.94	0.097	0.214	**-0.79**	**-0.169**	**0.033**	0.70	0.110	0.304	0.43	0.111	0.336	-0.84	-0.289	0.365	0.73	0.222	0.065
Gstm2	0.89	0.491	0.306	-0.39	N/A	0.383	0.15	N/A	0.847	0.47	0.307	0.292	-0.72	-0.591	0.485	-0.13	N/A	0.778
Gstp1	-0.85	-0.049	0.355	-0.31	N/A	0.493	0.68	0.168	0.320	-0.50	-0.089	0.250	-0.64	-0.212	0.559	0.23	N/A	0.614
Gucy1b3	0.59	0.014	0.599	-0.35	N/A	0.446	-0.51	-0.089	0.490	0.02	N/A	0.963	-0.94	-0.195	0.212	-0.01	N/A	0.975
H2-t23	0.67	0.143	0.535	-0.40	-0.058	0.373	0.92	0.091	0.076	0.24	N/A	0.610	-0.47	-0.133	0.687	0.74	0.174	0.055
Hdac2	-0.96	-0.039	0.177	0.23	N/A	0.615	-0.75	-0.025	0.246	-0.25	N/A	0.582	**-1.00**	**-0.017**	**0.014**	**-0.92**	**-0.031**	**0.004**
Hdac6	0.81	0.026	0.403	0.28	N/A	0.541	-0.18	N/A	0.819	0.32	N/A	0.490	0.99	0.279	0.090	-0.11	N/A	0.814
Hgf	0.81	0.158	0.398	-0.50	-0.139	0.254	0.30	N/A	0.696	0.32	N/A	0.481	-0.96	-0.143	0.183	0.22	N/A	0.628
Hif1a	-0.06	-0.002	0.960	0.10	N/A	0.834	-0.86	-0.033	0.136	0.09	N/A	0.852	-0.63	-0.021	0.569	0.71	0.041	0.073
Hmox1	0.99	0.160	0.108	-0.52	-0.164	0.236	0.46	0.224	0.544	0.24	N/A	0.604	-0.03	N/A	0.981	-0.37	N/A	0.409
Hspb1	0.92	0.040	0.260	-0.25	N/A	0.585	0.54	0.210	0.459	0.54	0.171	0.208	0.42	0.256	0.724	-0.34	N/A	0.452
Htra2	0.19	0.007	0.880	-0.21	N/A	0.657	-0.66	-0.023	0.342	-0.49	-0.022	0.265	**-1.00**	**-0.120**	**0.012**	0.33	N/A	0.473
Idh1	0.99	0.055	0.110	-0.25	N/A	0.595	0.33	N/A	0.671	-0.14	N/A	0.771	-0.83	-0.278	0.377	0.40	0.043	0.372
Il2rg	0.64	0.092	0.561	-0.48	-0.149	0.280	0.14	N/A	0.858	0.33	N/A	0.467	-0.34	N/A	0.781	-0.37	N/A	0.407
Il6	-0.37	-0.022	0.756	**0.84**	**0.263**	**0.018**	0.81	0.081	0.188	-0.11	N/A	0.808	-0.93	-0.244	0.239	-0.47	-0.070	0.292
Ins2	-0.76	-0.046	0.449	-0.73	-0.299	0.062	-0.08	N/A	0.918	-0.29	N/A	0.527	0.10	N/A	0.933	**-0.93**	**-0.296**	**0.002**
Ipcef1	-0.93	-0.125	0.236	0.39	N/A	0.392	0.58	0.028	0.417	-0.66	-0.099	0.109	**-1.00**	**-0.084**	**2.E-04**	0.10	N/A	0.839
Irak4	0.94	0.125	0.214	0.07	N/A	0.884	0.01	N/A	0.986	0.21	N/A	0.648	0.14	N/A	0.909	-0.61	-0.105	0.145
Irf7	0.98	0.059	0.123	-0.52	-0.329	0.226	0.92	0.299	0.083	0.31	N/A	0.497	-0.31	N/A	0.800	0.53	0.220	0.218
Itgam	0.95	0.286	0.201	**-0.76**	**-0.157**	**0.049**	-0.18	N/A	0.821	0.50	0.347	0.252	0.21	N/A	0.863	-0.30	N/A	0.512
Jun	**1.00**	**0.092**	**0.006**	-0.31	N/A	0.498	-0.01	N/A	0.986	0.37	N/A	0.416	0.38	N/A	0.749	-0.11	N/A	0.812
Keap1	0.23	N/A	0.851	-0.09	N/A	0.848	-0.66	-0.005	0.336	-0.70	-0.021	0.078	0.97	0.247	0.147	-0.36	N/A	0.421
Lilrb4a	0.95	0.773	0.199	-0.40	N/A	0.377	0.22	N/A	0.775	0.61	0.672	0.150	0.36	N/A	0.768	-0.25	N/A	0.593
Lpo	-0.08	N/A	0.949	**-0.84**	**-0.150**	**0.019**	0.57	0.068	0.427	-0.14	N/A	0.769	**1.00**	**0.168**	**0.009**	-0.56	-0.166	0.188
Lrrk2	-0.98	-0.042	0.111	0.04	N/A	0.928	0.10	N/A	0.900	0.37	N/A	0.419	0.81	0.081	0.403	0.15	N/A	0.754
Mapt	-0.67	-0.019	0.535	0.35	N/A	0.436	-0.38	N/A	0.621	**-0.80**	**-0.023**	**0.030**	-0.65	-0.067	0.552	0.54	0.025	0.207
Mgmt	0.94	0.119	0.222	-0.57	-0.115	0.178	-0.11	N/A	0.893	0.31	N/A	0.498	-0.13	N/A	0.916	-0.25	N/A	0.588
Mmp12	0.80	0.335	0.412	-0.32	N/A	0.481	0.30	N/A	0.701	0.21	N/A	0.654	0.40	N/A	0.740	0.50	0.171	0.252
Mmp14	0.97	0.278	0.156	-0.50	-0.331	0.258	0.40	0.175	0.597	0.41	0.188	0.366	-0.29	N/A	0.811	-0.11	N/A	0.814
Mpeg1	0.95	0.208	0.205	-0.56	-0.065	0.188	-0.06	N/A	0.944	-0.06	N/A	0.903	-0.12	N/A	0.924	-0.28	N/A	0.543
Mutyh	**1.00**	**0.045**	**0.023**	0.30	N/A	0.517	-0.01	N/A	0.991	-0.51	-0.050	0.244	**1.00**	**0.399**	**0.011**	0.08	N/A	0.857
Ncf1	0.99	0.225	0.109	-0.60	-0.144	0.152	0.14	N/A	0.862	0.23	N/A	0.625	0.46	0.201	0.695	-0.37	N/A	0.413
Nefh	-0.95	-0.100	0.201	0.62	0.125	0.141	0.68	0.078	0.322	-0.25	N/A	0.586	-0.50	-0.087	0.667	-0.53	-0.104	0.223
Nfe2l2	0.87	0.096	0.332	-0.51	-0.078	0.241	0.17	N/A	0.835	0.40	0.073	0.368	-0.88	-0.499	0.320	-0.15	N/A	0.752
Ngfg	0.74	0.114	0.467	0.63	0.216	0.128	-0.18	N/A	0.823	0.28	N/A	0.541	-0.84	-0.515	0.362	0.17	N/A	0.712
Ngfr	-0.98	-0.273	0.139	0.17	N/A	0.717	0.32	N/A	0.677	-0.53	-0.167	0.224	-0.14	N/A	0.911	0.44	0.179	0.326
Nme5	-0.99	-0.049	0.108	-0.28	N/A	0.536	-0.22	N/A	0.776	-0.28	N/A	0.543	0.93	0.160	0.247	0.29	N/A	0.524
Nol3	-0.65	-0.059	0.551	-0.51	-0.054	0.237	0.44	0.042	0.559	0.01	N/A	0.979	-0.92	-0.240	0.252	-0.21	N/A	0.652

	NeuroNexus						MARE						MA					
	Acute			Chronic			Acute			Chronic			Acute			Chronic		
Gene	r	s	p	r	s	p	r	s	p	r	s	p	r	s	p	r	s	p
Nos1	0.84	0.090	0.364	0.23	N/A	0.626	0.04	N/A	0.958	0.15	N/A	0.745	-0.80	-0.143	0.412	-0.15	N/A	0.756
Nos3	1.00	0.073	0.054	0.57	0.048	0.181	-0.21	N/A	0.787	0.15	N/A	0.748	0.83	0.109	0.379	**-0.85**	**-0.128**	**0.016**
Noxa1	0.81	0.023	0.396	0.59	0.095	0.161	0.00	N/A	0.998	-0.29	N/A	0.534	0.82	0.227	0.388	-0.04	N/A	0.937
Nqo1	**1.00**	**0.090**	**0.010**	-0.71	-0.086	0.076	0.10	N/A	0.903	0.60	0.061	0.153	-0.42	-0.112	0.722	-0.60	-0.075	0.155
Nr2f6	0.83	0.065	0.380	-0.27	N/A	0.552	-0.04	N/A	0.961	**0.76**	**0.069**	**0.046**	**-1.00**	**-0.228**	**0.031**	-0.32	N/A	0.487
Nr4a2	-0.62	-0.151	0.573	0.26	N/A	0.572	0.85	0.133	0.152	-0.50	-0.201	0.252	0.99	0.064	0.071	0.34	N/A	0.460
Osgin1	0.99	0.064	0.096	-0.43	-0.199	0.335	0.44	0.127	0.558	-0.02	N/A	0.972	0.11	N/A	0.929	0.05	N/A	0.921
Osmr	0.81	0.146	0.401	**-0.78**	**-0.090**	**0.037**	0.72	0.190	0.276	0.44	0.121	0.329	-0.40	-0.168	0.735	-0.17	N/A	0.720
Oxr1	-0.92	-0.033	0.256	0.38	N/A	0.398	-0.04	N/A	0.956	-0.14	N/A	0.769	-0.95	-0.253	0.201	0.55	0.043	0.200
Park7	-0.38	N/A	0.755	-0.22	N/A	0.635	-0.38	N/A	0.625	0.21	N/A	0.652	0.96	0.117	0.183	-0.26	N/A	0.570
Parp1	-0.30	N/A	0.809	0.27	N/A	0.553	-0.70	-0.036	0.295	0.65	0.035	0.117	0.98	0.051	0.129	0.06	N/A	0.899
Pdgfrb	0.95	0.141	0.206	-0.56	-0.120	0.189	0.20	N/A	0.804	0.10	N/A	0.824	-0.49	-0.192	0.677	0.14	N/A	0.767
Pink1	0.77	0.033	0.438	0.57	0.017	0.185	-0.67	-0.014	0.331	-0.18	N/A	0.701	**-1.00**	**-0.151**	**0.024**	0.02	N/A	0.972
Pla2g4a	**1.00**	**0.099**	**0.023**	-0.74	-0.115	0.057	0.01	N/A	0.992	0.41	0.135	0.366	-0.74	-0.231	0.471	-0.27	N/A	0.556
Ppargc1a	-0.85	-0.028	0.357	0.11	N/A	0.819	-0.16	N/A	0.836	-0.10	N/A	0.826	0.40	0.068	0.738	0.07	N/A	0.888
Prnp	**-1.00**	**-0.042**	**0.037**	-0.13	N/A	0.788	-0.39	N/A	0.606	-0.53	-0.029	0.217	**-1.00**	**-0.168**	**0.011**	0.64	0.017	0.121
Psen1	-0.78	-0.025	0.431	0.13	N/A	0.782	-0.79	-0.023	0.212	0.31	N/A	0.504	-0.99	-0.044	0.067	-0.33	N/A	0.474
Psmb8	0.87	0.165	0.324	-0.69	-0.259	0.084	0.47	0.176	0.528	0.29	N/A	0.528	0.17	N/A	0.892	-0.18	N/A	0.704
Ptgs2	-0.95	-0.078	0.202	0.05	N/A	0.909	-0.62	-0.206	0.380	0.60	0.124	0.153	0.37	N/A	0.757	0.18	N/A	0.696
Ptpn6	0.98	0.102	0.142	-0.45	-0.045	0.317	0.20	N/A	0.803	0.47	0.173	0.290	0.53	0.158	0.642	-0.51	-0.076	0.242
Ptx3	-0.61	-0.137	0.585	-0.53	-0.130	0.218	0.73	0.223	0.270	0.00	N/A	0.992	-0.33	N/A	0.789	-0.36	N/A	0.424
Rela	0.90	0.028	0.286	-0.48	-0.053	0.274	0.29	N/A	0.713	-0.23	N/A	0.625	0.16	N/A	0.896	-0.17	N/A	0.717
Scd1	0.95	0.118	0.211	-0.03	N/A	0.950	0.59	0.071	0.406	0.25	N/A	0.590	-0.99	-0.181	0.080	-0.37	N/A	0.418
Serpina3n	0.61	0.150	0.585	-0.36	N/A	0.434	-0.37	N/A	0.632	0.34	N/A	0.458	-0.29	N/A	0.812	-0.47	-0.481	0.283
Sirt1	0.90	0.063	0.281	-0.65	-0.038	0.116	0.03	N/A	0.972	0.19	N/A	0.688	0.70	0.042	0.510	0.60	0.059	0.157
Sirt2	-0.89	-0.043	0.301	-0.47	-0.033	0.289	0.67	0.051	0.328	-0.26	N/A	0.570	-0.94	-0.250	0.219	-0.15	N/A	0.750
Slc8a1	-0.79	-0.052	0.419	0.57	0.047	0.177	0.76	0.061	0.241	-0.50	-0.055	0.256	-0.97	-0.059	0.146	0.62	0.062	0.136
Snca	-0.33	N/A	0.783	0.27	N/A	0.558	-0.20	N/A	0.797	-0.63	-0.043	0.132	0.81	0.201	0.398	0.47	0.050	0.283
Sod1	0.80	0.037	0.408	-0.16	N/A	0.728	0.04	N/A	0.959	-0.29	N/A	0.525	**-1.00**	**-0.270**	**3.E-04**	0.11	N/A	0.807
Sod2	0.79	0.008	0.416	0.29	N/A	0.524	-0.35	N/A	0.649	-0.03	N/A	0.942	-0.88	-0.089	0.316	-0.34	N/A	0.451
Sod3	0.93	0.038	0.240	0.22	N/A	0.635	-0.76	-0.051	0.245	0.42	0.041	0.342	0.41	0.048	0.729	**-0.79**	**-0.107**	**0.033**
Spp1	**1.00**	**0.475**	**0.013**	-0.16	N/A	0.738	0.24	N/A	0.757	**0.79**	**0.799**	**0.036**	0.53	0.742	0.647	-0.36	N/A	0.422
Src	0.81	0.063	0.400	-0.13	N/A	0.776	-0.66	-0.104	0.344	0.63	0.063	0.131	0.92	0.151	0.263	-0.10	N/A	0.826
Srxn1	0.99	0.126	0.085	0.17	N/A	0.721	-0.22	N/A	0.777	-0.12	N/A	0.801	0.93	0.052	0.246	-0.08	N/A	0.871
Stx2	0.80	0.045	0.414	0.37	N/A	0.411	0.44	0.024	0.563	-0.29	N/A	0.523	-0.98	-0.276	0.125	0.18	N/A	0.702
Tnf	0.67	0.098	0.531	-0.62	-0.202	0.137	0.77	0.139	0.231	0.20	N/A	0.667	0.93	0.313	0.241	0.08	N/A	0.861
Tnfrsf1a	0.84	0.132	0.369	-0.52	-0.120	0.237	0.24	N/A	0.763	0.49	0.139	0.263	-0.46	-0.211	0.692	-0.29	N/A	0.527
Tnfrsf25	0.86	0.102	0.345	0.26	N/A	0.572	**0.96**	**0.060**	**0.036**	0.15	N/A	0.747	0.98	0.376	0.114	-0.57	-0.062	0.178
Tor1a	**1.00**	**0.046**	**0.029**	-0.43	-0.021	0.330	-0.11	N/A	0.890	**-0.77**	**-0.029**	**0.045**	**-1.00**	**-0.208**	**0.015**	0.66	0.023	0.110
Tpm1	0.58	0.014	0.606	-0.18	N/A	0.697	0.94	0.038	0.055	-0.20	N/A	0.668	0.88	0.154	0.319	0.12	N/A	0.805
Trp53	0.84	0.072	0.370	-0.43	-0.024	0.333	-0.12	N/A	0.882	0.36	N/A	0.429	-0.77	-0.205	0.439	0.08	N/A	0.863
Trpm2	0.29	N/A	0.812	0.08	N/A	0.869	0.34	N/A	0.664	0.07	N/A	0.878	0.97	0.075	0.166	-0.40	N/A	0.377
Txnl1	-0.99	-0.021	0.089	0.31	N/A	0.500	-0.39	N/A	0.606	-0.60	-0.023	0.154	0.71	0.050	0.499	0.59	0.027	0.161
Txnrd1	1.00	0.012	0.052	0.16	N/A	0.731	-0.39	N/A	0.612	-0.33	N/A	0.470	-0.89	-0.094	0.307	0.36	N/A	0.426
Tyropb	**1.00**	**0.194**	**0.025**	-0.60	-0.118	0.157	0.19	N/A	0.807	0.31	N/A	0.498	0.33	N/A	0.787	-0.38	N/A	0.394
Ubqln1	-0.96	-0.045	0.187	0.43	0.020	0.338	-0.65	-0.031	0.352	-0.09	N/A	0.848	0.89	0.119	0.305	0.47	0.019	0.291
Vegfa	0.42	0.037	0.725	0.35	N/A	0.438	-0.78	-0.070	0.221	0.37	N/A	0.407	-0.64	-0.089	0.561	-0.36	N/A	0.432
Xbp1	0.72	0.045	0.488	-0.58	-0.029	0.168	0.79	0.018	0.206	-0.02	N/A	0.960	0.96	0.141	0.178	-0.44	-0.015	0.319
